# Derlin rhomboid pseudoproteases employ substrate engagement and lipid distortion to enable the retrotranslocation of ERAD membrane substrates

**DOI:** 10.1016/j.celrep.2021.109840

**Published:** 2021-10-19

**Authors:** Anahita Nejatfard, Nicholas Wauer, Satarupa Bhaduri, Adam Conn, Saroj Gourkanti, Narinderbir Singh, Tiffany Kuo, Rachel Kandel, Rommie E. Amaro, Sonya E. Neal

**Affiliations:** 1 Division of Biological Sciences, Section of Cell and Developmental Biology, University of California San Diego, La Jolla, CA 92093, USA; 2 Department of Chemistry and Biochemistry, University of California San Diego, La Jolla, CA 92093, USA; 3 Lead contact

## Abstract

Nearly one-third of proteins are initially targeted to the endoplasmic reticulum (ER) membrane, where they are correctly folded and then delivered to their final cellular destinations. To prevent the accumulation of misfolded membrane proteins, ER-associated degradation (ERAD) moves these clients from the ER membrane to the cytosol, a process known as retrotranslocation. Our recent work in *Saccharomyces cerevisiae* reveals a derlin rhomboid pseudoprotease, Dfm1, is involved in the retrotranslocation of ubiquitinated ERAD membrane substrates. In this study, we identify conserved residues of Dfm1 that are critical for retrotranslocation. We find several retrotranslocation-deficient Loop 1 mutants that display impaired binding to membrane substrates. Furthermore, Dfm1 possesses lipid thinning function to facilitate in the removal of ER membrane substrates, and this feature is conserved in its human homolog, Derlin-1, further implicating that derlin-mediated retrotranslocation is a well-conserved process.

## In brief

ER-associated degradation is a conserved pathway of protein quality control that requires the retrotranslocation of ubiquitinated substrates from the ER to the cytoplasm for degradation. Nejatfard et al. show that derlin rhomboid pseudoproteases mediate the retrotranslocation of misfolded membrane substrates via a mechanism that is conserved from yeast to humans.

## Graphical abstract

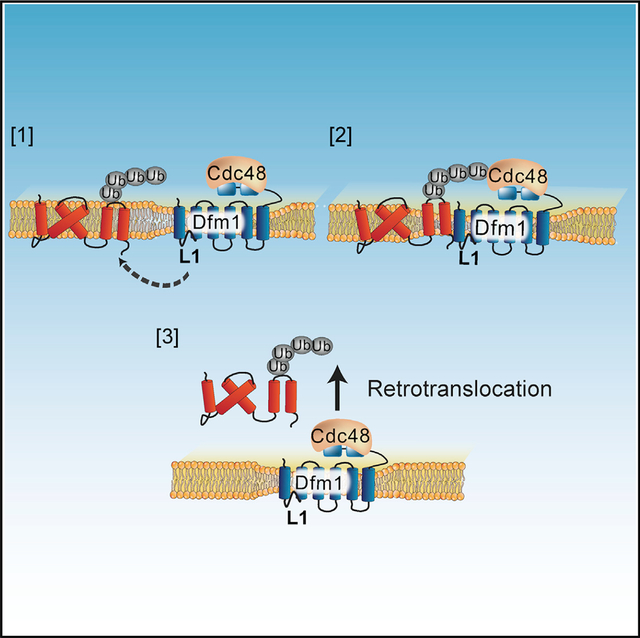

## INTRODUCTION

Almost all eukaryotic membrane and secreted proteins, comprising one-third of the eukaryotic proteome, are co-translationally imported into the endoplasmic reticulum (ER), where they are subsequently folded (Wang and Dehesh, 2018; Sicari et al., 2019). Often, proteins fail to fold or assemble properly, at which point they are eliminated by ER-associated degradation (ERAD) (Ruggiano et al., 2014; Mehrtash and Hochstrasser, 2019; Sun and Brodsky, 2019). ERAD is a highly conserved quality-control pathway that involves several key steps (Hampton and Garza, 2009; Vashistha et al., 2016; Mehrtash and Hochstrasser, 2019): (1) recognition of misfolded ER proteins; (2) polyubiquitination of substrates by one or more E3 ligases; (3) movement or extraction of ER substrates to the cytosol (termed retrotranslocation), which is powered by Cdc48/p97 AAA-ATPase (ATPases Associated with Diverse Cellular Activity); and (4) degradation by the cytosolic proteasome. ERAD recognizes different classes of substrates based on the location of the lesion within the protein. Substrates for ERAD include misfolded soluble luminal proteins (ERAD-L) and integral membrane proteins with lesions in their transmembrane domain (ERAD-M) (Hampton et al., 1996; Plemper et al., 1998; Vashist and Ng, 2004; Wangeline and Hampton, 2018) or their cytosolic domain (ERAD-C) (Ravid et al., 2006). The HRD (Hydroxymethyl glutaryl-coenzyme A reductase degradation) pathway uses E3 ubiquitin ligase Hrd1 for ubiquitination of both ERAD-M and ERAD-L substrates, whereas the DOA (Degradation of alpha 2) pathway uses E3 ubiquitin ligase Doa10 for ubiquitination of ERAD-C substrates (Laney and Hochstrasser, 2003; Carvalho et al., 2006; Chen et al., 2006; Hampton and Garza, 2009; Foresti et al., 2013).

A common feature of all ERAD pathways is the requirement for moving substrates from the ER to the cytosol for degradation, a process known as retrotranslocation (Hampton and Sommer, 2012). Despite intense studies on this pathway (Garza et al., 2009; Peterson et al., 2019; Schmidt et al., 2020; Wu et al., 2020), the protein or “exit channel” required for retrotranslocating multi-spanning membrane substrates remained unknown for over two decades and has been only recently identified. By screening a complete collection of yeast mutants via SPOCK (single-plate orf compendium kit), we pinpointed yeast derlin, Dfm1, as a specific mediator for the retrotranslocation of multispanning membrane substrates in ERAD (Neal et al., 2018). This finding contradicted previous results in which Dfm1 had either a partial or no role in ERAD (Sato and Hampton, 2006; Goder et al., 2008; Stolz et al., 2010; Avci et al., 2014). We resolved this discrepancy by finding that loss of Dfm1, along with strong expression of membrane substrates, imposes a growth stress on cells and induces HRD complex remodeling to restore ERAD-M retrotranslocation (Neal et al., 2018, 2020).

Sequence and structural homology indicate that derlins belong to the rhomboid superfamily (Greenblatt et al., 2011). The rhomboid superfamily is known for their many roles in diverse membrane-related processes (Düsterhöft et al., 2017; Kandel and Neal, 2020). Many rhomboid proteins are intramembrane proteases and typically cleave membrane substrates within the lipid bilayer via the serine-histidine dyad active site (Lemieux et al., 2007; Bondar et al., 2009; Zhou et al., 2012; Uritsky et al., 2016; Shokhen and Albeck, 2017; Tichá et al., 2018). There is a large subclass of the rhomboid superfamily that lacks residues for proteolysis and is known as rhomboid pseudoproteases (Lemberg and Freeman, 2007; Lemberg and Adrain, 2016; Began et al., 2020). Remarkably, rhomboid pseudoproteases have conserved rhomboid residues, implying the intriguing idea that derlins utilize rhomboid features for executing retrotranslocation functions. Bacterial rhomboid proteases’ compact architectural fold is presumed to induce local perturbations of the lipid bilayer for gaining substrate access prior to cleavage (Wang et al., 2006; Lemieux et al., 2007; Bondar et al., 2009; Moin and Urban, 2012; Brooks and Lemieux, 2013). An intriguing possibility is that Dfm1 has retained membrane-perturbing properties of its bacterial counterpart to facilitate the movement of substrates across the membrane. In support of this idea, Dfm1’s yeast paralog, Der1, has been shown to induce lipid thinning to assist in the retrotranslocation of ER luminal substrates (Wu et al., 2020). However, it is yet to be determined whether the same mechanism is applied for the removal of integral membrane proteins from the ER.

In the studies below, we have explored these questions by performing a non-biased sequence analysis of Dfm1 coupled with cell biological assays and computational simulations to characterize the mechanistic features associated with retrotranslocation. Herein, we identified a subset of retrotranslocation-deficient mutants that are enriched in Loop 1 (L1) and transmembrane domain 2 (TM2) regions of Dfm1. Closer analysis reveals L1 retrotranslocation-deficient mutants are unable to bind to ERAD membrane substrates, indicating a role for L1 region in substrate detection. Furthermore, molecular dynamics (MD) simulations on Dfm1 homology model revealed that Dfm1 possesses membrane lipid thinning function. Indeed, retrotranslocation-deficient mutants are localized at TM2, which is critical for lipid thinning as delineated by computational modeling. Notably, Dfm1 retrotranslocation-deficient mutants are located at sites that are highly conserved in mammalian derlins. We sought to determine the conservation of Dfm1’s mechanism and found that a subset of Dfm1 sites identified from our screen also contribute to the membrane substrate binding, ERAD, and lipid thinning function of human derlin, Derlin-1. Derlin-1 has been previously shown to utilize its rhomboid features for retrotranslocation and is implicated in several pathologies, including cancer, cystic fibrosis, neuropathies, and viral infection (Greenblatt et al., 2011; Kandel and Neal, 2020). Overall, our study sheds light on how derlin rhomboid pseudoproteases have evolved to carry out the critical and widely conserved process of membrane protein quality control.

## RESULTS

### Yeast Dfm1 has highly conserved rhomboid and derlin-specific residues

Sequence alignment of Dfm1, along with other members of the rhomboid superfamily, reveals Dfm1 contains residues that are highly conserved across the rhomboid superfamily (Figure S1A, highlighted in red and yellow). Along with having common rhomboid features, Dfm1 has conserved residues that are specifically retained in mammalian derlin rhomboid pseudoproteases (Figure S1A, encircled in orange). In this study, we sought to understand the extent that rhomboid- and/or derlin-specific features of Dfm1 are required for membrane substrate retrotranslocation.

### Rhomboid WR and GxxxG are not sufficient for retrotranslocation

**Figure 1.**
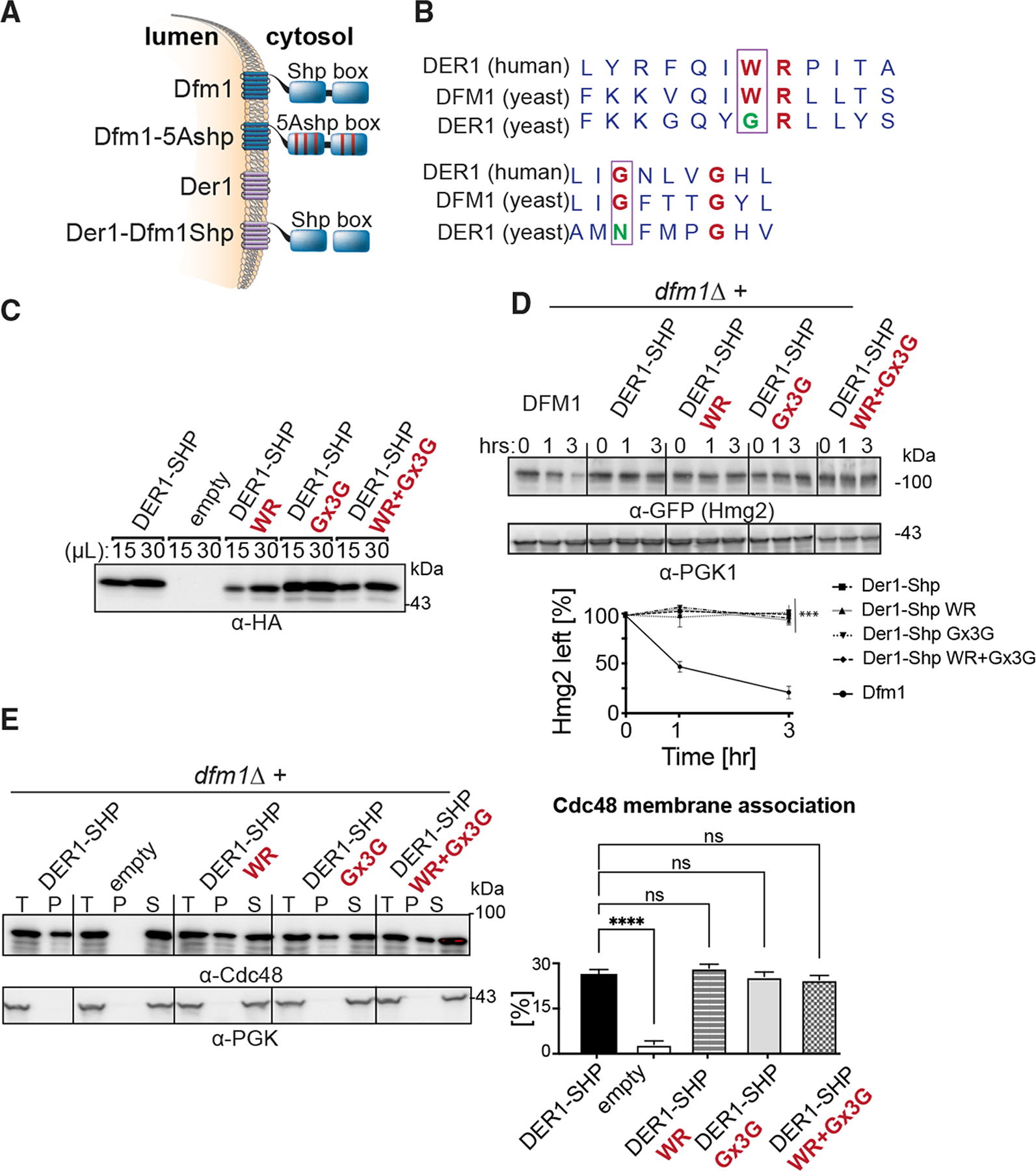
WR and GxxxG motifs are not sufficient for ERAD-M retrotranslocation (A) Depiction of Dfm1, Der1, and Der1-Shp. Dfm1 and Der1 are ER-localized membrane proteins with six transmembrane domains. (B) Alignment of WR and GxxxG motif from *H. sapiens* Derlin-1 and *S. cerevisiae* Der1 and Dfm1. (C) Expression levels of Der1-Shp variants are measured by loading increasing amounts of lysates (15 and 30 μL) on SDS-PAGE followed by immunoblotting with α-HA. (D) In the indicated strains, degradation of Hmg2-GFP was measured by CHX-chase assay. Cells were analyzed by SDS-PAGE and immunoblotted for Hmg2-GFP with α-GFP. Band intensities were normalized to PGK1 loading control and quantified by ImageJ. t = 0 was taken as 100%, and data are represented as mean ± SEM from n = 3 biological replicates, ***p < 0.001, repeated-measures ANOVA. (E) Total cell lysates (T) from the indicated strains were separated into soluble cytosolic fraction (S) and pellet microsomal fraction (P) upon centrifugation at 14,000 × *g*. Each fraction was analyzed by SDS-PAGE and immunoblotted for Cdc48 with α-Cdc48 and Pgk1 with α-Pgk1. The graph shows the quantification of Cdc48 in the pellet fractions of the respective cells as measured from ImageJ. Data are represented as percentage of Cdc48 that is bound to pellet fraction and is shown as mean ± SEM from n = 3 biological replicates, ****p < 0.0001, one-way ANOVA.

Dfm1 contains the highly conserved WR motif in L1 and a GxxxG motif in TM6. We and others have shown that both motifs are important for rhomboid derlin retrotranslocation function (Greenblatt et al., 2011; Neal et al., 2018). We examined whether the WR and GxxxG motifs are sufficient for Dfm1 retrotranslocation function through use of chimeras. We used the Der1-SHP chimera from our previous studies, which consists of Dfm1’s closest homolog, Der1, fused to Dfm1’s cytoplasmic SHP tail (Figure 1A) (Neal et al., 2018). Our previous work indicated that Der1-SHP supports Cdc48 recruitment via binding of Cdc48 to the chimera’s SHP tail, but does not support retrotranslocation through Der1’s transmembrane segment (Figure 1A) (Neal et al., 2018). A closer examination of Der1’s transmembrane regions shows that Der1 does not possess the highly conserved WR and GxxxG motif and harbors GR and NxxxG instead (Figure 1B). To determine whether the WR and GxxxG motifs are sufficient in supporting Der1-Shp retrotranslocation function, we inserted both motifs at homologous sites within the Der1-Shp transmembrane region. Three mutants were generated: Der1-Shp-WR, Der1-Shp-GxxxG, and Der1-Shp-WR+GxxxG. All three Der1-Shp mutants had the same stability as the unaltered Der1-Shp chimera protein and were still able to support Cdc48 recruitment as indicated with a direct assay of Cdc48 binding to the microsomal pellet (Figures 1C–1E). Despite this, neither mutant could facilitate degradation of Hmg2-GFP through ERAD, implying additional sequence residues within Dfm1’s transmembrane segments are required for retrotranslocation.

### Dfm1 L1 and TM2 mutants are unable to retrotranslocate ER membrane substrates

**Figure 2.**
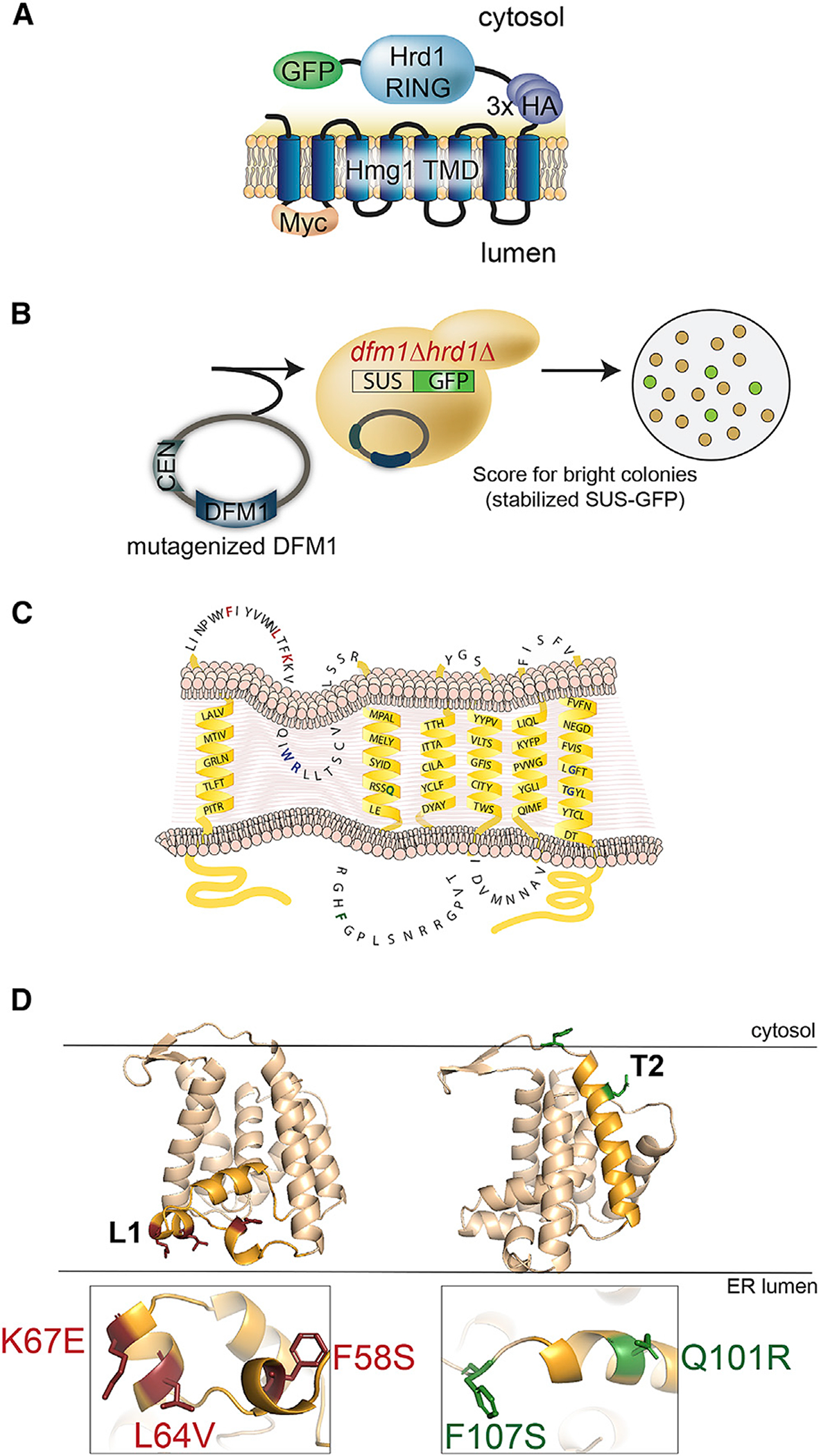
Dfm1 is intolerable to mutations in Loop 1 (L1) and transmembrane domain 2 (TM2) (A) Depiction of fusion protein, SUS-GFP. The transmembrane Hmg1 domain has a luminal Myc epitope and cytosolic 3×HA epitopes fused to the catalytic Hrd1 RING. (B) Mutagenized DFM1 was transformed into *dfm1*Δ *hrd1*Δ cells expressing SUS-GFP and scored for stabilization of SUS-GFP or high colony fluorescence by visualization. (C) Depiction of Dfm1 mutants (indicated in red for L1 and green for TM2) that were selected from the random mutagenesis screen and validated for expression, localization to ER, and Cdc48 recruitment function. (D) Homology model of Dfm1. Positions of L1 and TM2 mutants are indicated in red and green, respectively.

To identify additional residues required for Dfm1’s retrotranslocation function, we performed a sequence analysis screen in which random mutagenesis was performed on Dfm1’s transmembrane segment. We excluded mutagenic alteration of the cytoplasmic SHP tail region to prevent false positives caused by the disruption of Dfm1’s Cdc48 recruitment function. Dfm1 was mutagenized by using GeneMorph II random mutagenesis kit. Mutagenized Dfm1 was introduced to *dfm1*Δ *hrd1*Δ null yeast cells containing an optical, self-ubiquitinating substrate, SUS-GFP, a substrate used in our previous screen for discovery and study of Dfm1 retrotranslocation (Figures 2A and 2B) (Neal et al., 2018). Notably, because Hrd1 has been shown to be required for restoring retrotranslocation function when Dfm1 is absent (Neal et al., 2018, 2020), our screening strain also has Hrd1 missing to prevent suppression of strains during the random mutagenesis screen. The resulting transformants were screened for high colony fluorescence as a result of buildup of SUS-GFP, indicating Dfm1 loss of function and inability to retrotranslocate SUS-GFP. Plasmids were extracted from yeast transformants exhibiting high fluorescence and sequenced to discern the causative Dfm1 mutations. Dfm1 mutants containing no premature stop codons and harboring a single mutation were subjected to further analysis. Less interesting possibilities for the loss of Dfm1 function include low expression, incorrect cellular localization, or inability to recruit Cdc48. Accordingly, successful Dfm1 candidate mutants were analyzed for their stability, and if they were robustly expressed, they were then subjected to cellular fractionation and Cdc48 binding assays to exclude mutants that would not elucidate the mechanism of Dfm1. Random mutagenesis was performed near saturation because we were able to recover the same mutants within Dfm1’s TM region. Specifically, we recovered and identified mutants showing robust expression, correct ER localization, and no disruption in Cdc48 recruitment activity (Figures S1B–S1D). Dfm1 was also found to be associated with the E3 ligase HRD complex (Stolz et al., 2010). Through co-immunoprecipitation, we validated that all five mutants did not disrupt their association with major components of the HRD complex, which includes the E3 ligase Hrd1 and its partner protein, Hrd3 (Figure S1E). Next, we generated a structural model of Dfm1 using homology modeling on Der1, an isoform of Dfm1 whose structure has been solved (Wu et al., 2020). Based on this homology model, Dfm1 mutants were enriched in both L1 (F58S, L64V, and K67E) and TM2 (Q101R and F107S, respectively) (Figures 2C and 2D; Figure S1A, blue asterisks). Interestingly, K67 is conserved across the rhomboid family, whereas F58, L64, and F107 are conserved specifically among derlins (Figure S1A).

### L1 and TM2 mutants affect retrotranslocation and ERAD of ER membrane substrates

**Figure 3.**
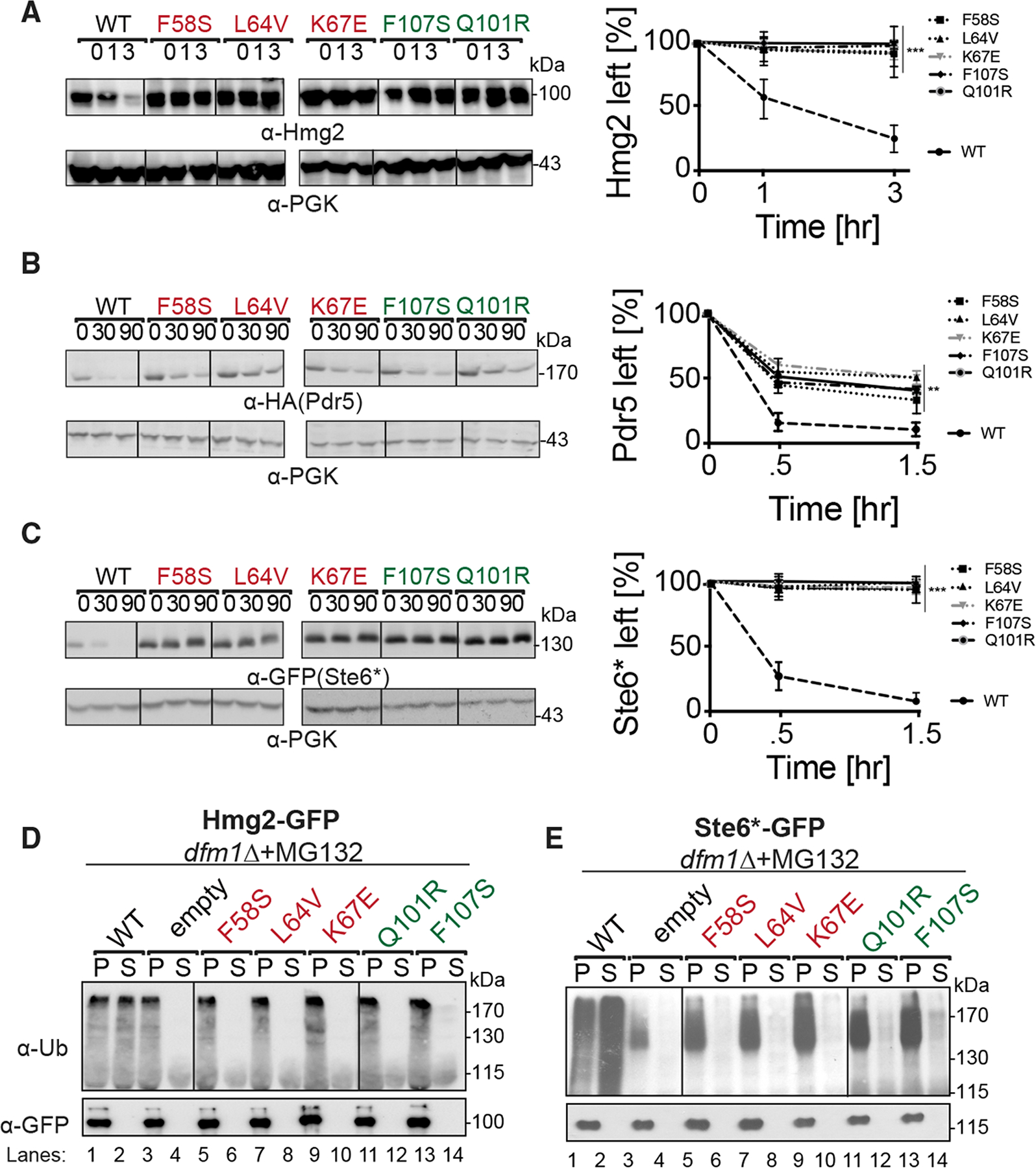
Dfm1 mutants are defective in ERAD-M degradation and retrotranslocation (A) *dfm1*Δ strains harboring the indicated DFM1 mutants were grown to log-phase, and ERAD-M degradation was measured by CHX chase, analyzed by SDS-PAGE, and immunoblotted for Hmg2-GFP with α-GFP. (B) Same as (A), expect degradation of Pdr5*-HA was measured. (C) Same as (A), expect degradation of Ste6*-GFP was measured. (D) Crude lysates from each strain were ultracentrifuged to discern ubiquitinated Hmg2-GFP that either has been retrotranslocated into the soluble fraction (S) or remained in the membrane (P). Following fractionation, Hmg2-GFP was immunoprecipitated from both fractions, resolved on 8% SDS-PAGE, and immunoblotted with α-GFP and α-Ubi. (E) Same as (D), except *in vivo* retrotranslocation assay was performed on Ste6*-GFP. For (A)–(C), band intensities were normalized to PGK1 loading control and quantified by ImageJ. t = 0 was taken as 100%, and data are represented as mean ± SEM from n = 3 biological replicates, **p < 0.01, ***p < 0.001, repeated-measures ANOVA.

We examined the extent to which the Dfm1 mutants affected ERAD of various substrates. Test substrates included all Dfm1-dependent membrane substrates characterized in our previous studies: integral membrane HRD pathway substrates Hmg2 and Pdr5* and integral membrane DOA pathway substrate Ste6*. The effect of Dfm1 mutants was directly tested with cycloheximide (CHX)-chase assay on Hmg2, Pdr5*, and Ste6* (Figures 3A–3C). The normally degraded substrates, Hmg2 and Ste6*, were completely stabilized by each Dfm1 mutant. In contrast, we observed partial degradation of Pdr5* with each Dfm1 mutant. This is most likely due to the rapid suppressive nature of Dfm1 mutants, which we previously observed to be triggered by overexpression of several ERAD-M substrates (Neal et al., 2018, 2020; Bhaduri and Neal, 2021). Nevertheless, the Pdr5* degradation rate by Dfm1 mutants was significantly slower compared with Pdr5*degradation by wild-type Dfm1. Overall, Dfm1 mutants isolated from the genetic screen affect degradation of ER membrane substrates.

We confirmed that Hmg2 and Ste6* retrotranslocation was strongly blocked by all Dfm1 mutants in the *in vivo* retrotranslocation assay (Figures 3D and 3E). Cells with wild-type functional Dfm1 showed normal Hmg2 and Ste6* retrotranslocation, as indicated by buildup of ubiquitinated Hmg2 and Ste6* in the supernatant (S) fraction, as a result of inhibition of proteasome function by MG132. In contrast, identical expression of each Dfm1 mutant resulted in buildup of ubiquitinated Hmg2 and Ste6* in the microsomal pellet (P) fraction. This inhibition in retrotranslocation is comparable with control strains with Dfm1 absent. Thus, by all conditions examined, Dfm1 L1and L2/TM2 mutants are dysfunctional in ERAD and retrotranslocation of ER membrane substrates tested.

### Dfm1 binds specifically to membrane substrates and not luminal substrates

**Figure 4.**
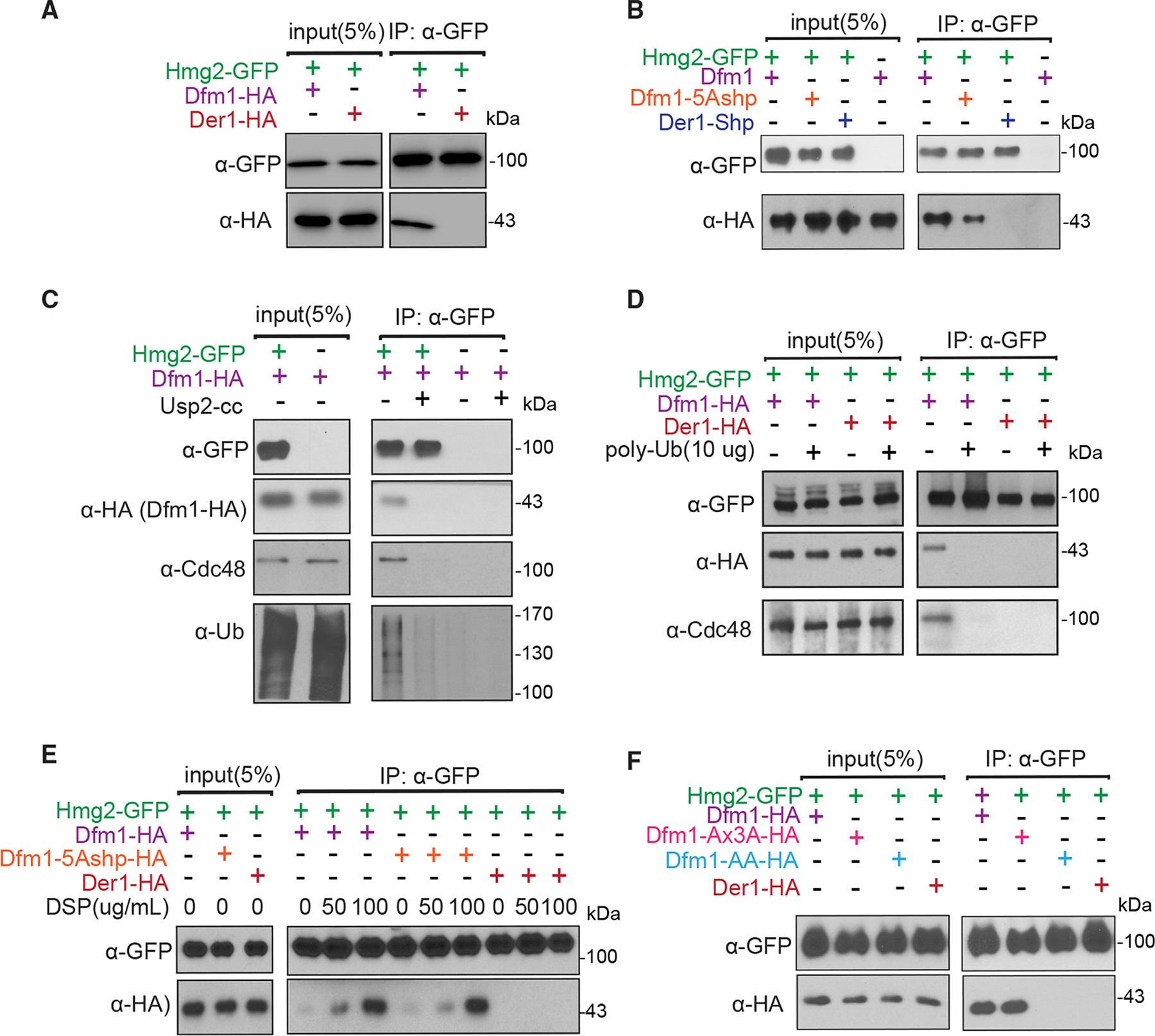
Dfm1’s WR motif and SHP box are required for interaction with membrane substrates (A) Hmg2-GFP and Dfm1-HA binding were analyzed by coIP. As a control for specificity, cells expressing Der1-HA were used. (B) Same as (A), except binding of Hmg2-GFP to Dfm1 variants, Dfm1-5Ashp and Der1-Shp, was analyzed. (C) Dfm1-Cdc48 complex interacts directly with the polyubiquitin chain of Hmg2. Microsomes isolated from indicated strains were treated with Usp2Core, and Hmg2-GFP was immunoprecipitated, resolved on 8% SDS-PAGE, and immunoblotted for ubiquitin with α-Ub, Hmg2-GFP with α-GFP, Cdc48 with α-Cdc48, and Dfm1 with α-HA. (D) Addition of Lys48-linked polyubiquitin chains disrupts binding of Hmg2 to Dfm1-Cdc48. Hmg2-GFP, Dfm1-HA, and Cdc48 binding were analyzed by coIP in the presence of an increasing amount of Lys48-linked polyubiquitin chains (2, 5, and 10 μg). As a negative control, strains not expressing Hmg2-GFP were used. (E) Crosslinking analysis of Hmg2-GFP and Dfm1-5Ashp. Microsomes were harvested from DSP-treated strains and subjected to immunoprecipitation of Hmg2-GFP with GFP Trap, followed by immunoblotting for Dfm1-5Ashp with anti-HA and Hmg2 with anti-GFP. (F) Same as (A), except binding to Hmg2-GFP was analyzed with Dfm1 variants: Dfm1-AA and Dfm1-Ax3A.

Our previous studies showed that Dfm1 is selective for retrotranslocating membrane substrates and not luminal substrates, such as CPY*, KHN, and KWW (Neal et al., 2018). This implied that Dfm1 selectively binds membrane substrates and not luminal substrates. To test this, we directly examined Dfm1 interaction with various classes of ERAD substrates. We analyzed Dfm1 interactions with its well-characterized integral membrane substrates: Hmg2, Pdr5*, and Ste6*. Hmg2-GFP, Pdr5*-Myc, and Ste6*-GFP were immunoprecipitated with GFP or Myc Trap antibodies from lysates of various strains co-expressing Dfm1-HA, followed by SDS-PAGE and immunoblotting for Dfm1 with (Hemagglutinin) α-HA, Hmg2, and Ste6* with α-GFP and Pdr5* with α-Myc (Figures 4A, S2A, and S2B). In all cases, binding of membrane substrates to Dfm1 was clear. As a control, we tested the very similar, but uninvolved, Der1 homolog for binding to Hmg2-GFP, Pdr5*-Myc, and Ste6*-GFP, and we found no association (Figures 4A, S2A, and S2B). We similarly tested Dfm1 interaction with a classical luminal ERAD-L substrate CPY*. CPY*-GFP was immunoprecipitated with GFP Trap antibodies from lysates co-expressing Dfm1-HA, followed by immunoblotting for Dfm1 with α-HA and CPY* with α-GFP. We did not observe an association of Dfm1 with CPY*, whereas an association was seen with Der1, its canonical substrate (Figure S2C). These results suggest Dfm1 interacts specifically with ER membrane substrates, but not luminal substrates.

### Shp tail is necessary, but not sufficient, for substrate binding

We have previously shown that alteration of the five signature residues of the Dfm1 SHP box to alanine (Dfm1-5Ashp) removed its ability to recruit Cdc48 (Figure 1A) (Sato and Hampton, 2006; Neal et al., 2018). Conversely, we have shown that addition of the Dfm1 SHP motif to the normally SHP-less Der1 made this chimera able to promote Cdc48 recruitment comparable with Dfm1, but it was not sufficient for supporting retrotranslocation (Figure 1A) (Neal et al., 2018). We tested these SHP variants for their ability to bind substrates Hmg2, Pdr5*, and Ste6*. Notably, the Dfm1-5Ashp mutant that failed to recruit Cdc48 displayed marked disruption in substrate binding with only a small fraction of Dfm1-5Ashp (<5%) bound to ERAD-M substrates Hmg2-GFP and Pdr5*-GFP and ERAD-C substrate Ste6*-GFP compared with wild-type Dfm1, suggesting requirement of SHP tail for substrate association (Figures 4B, S2A, and S2B). Furthermore, the Der1-SHP chimera that recruited Cdc48 could not support binding to Hmg2, Pdr5*, and Ste6*. Thus, the SHP tail, as well as presumably Cdc48 recruitment to the ER membrane, is necessary, but not sufficient, for substrate association.

### Dfm1 recruits Cdc48 to bind to polyubiquitin chains conjugated to membrane substrates

The requirement for Dfm1’s SHP motif suggests that Cdc48 is also involved with binding membrane substrates. Previously, we demonstrated that Dfm1 mediates Cdc48 recruitment to the ER surface (Neal et al., 2018). Moreover, we established that Cdc48 functions as a “retrochaperone” by directly binding to the polyubiquitin chain of membrane substrates to maintain solubility and prevent aggregation of substrates retrotranslocated into the aqueous cytosol (Neal et al., 2017). Based on these observations, we hypothesize that Dfm1 recruits Cdc48 and, concomitantly, that Cdc48 attaches to the polyubiquitin chains of membrane substrates targeted for retrotranslocation and degradation. We tested whether ubiquitin removal from membrane substrates causes dissociation of substrates from the Dfm1-Cdc48 complex. We used the Usp2Core ubiquitin protease to remove the multiubiquitin chain from ubiquitinated Hmg2-GFP embedded in microsomal membranes. Removal of the ubiquitin chain from Hmg2-GFP caused loss of both the covalently bound ubiquitin and the associated Dfm1-Cdc48 complex (Figure 4C). Because the Dfm1-Cdc48 complex binds polyubiquitin chains, we next tested the effect of adding commercially available free polyubiquitin chains on the association of Dfm1-Cdc48 complex with Hmg2-GFP. Direct addition of increasing amounts of Lys-48-linked polyubiquitin to microsome fractions completely blocked the association of Dfm1-Cdc48 with Hmg2-GFP as assessed by GFP-Trap coprecipitation (Figure 4D). We interpreted this to mean that ubiquitinated Hmg2 is removed by direct competition of free polyubiquitin chains.

### WR motif is required for membrane substrate binding

The above studies show that Dfm1’s SHP tail, through Cdc48 binding, is required for direct interaction with polyubiquitinated membrane substrates. We observed no association of membrane substrate with chimera Der1-SHP with intact Cdc48 recruitment function, suggesting Cdc48 is not sufficient for substrate interaction (Figure 4B). Furthermore, a small fraction of Dfm1-5Ashp without intact Cdc48 recruitment function was found to be associated to Hmg2-GFP, suggesting a transient interaction between Dfm1-5Ashp and Hmg2 (Figure 4B). Indeed, we observed stable association of Hmg2-GFP with Dfm1-5Ashp with crosslinking, confirming that the interaction is transient and suggests substrate binding is mediated by additional information within the Dfm1 transmembrane region and is independent of ubiquitin binding (Figure 4E). Accordingly, we analyzed whether additional residues within Dfm1’s transmembrane domains are required for membrane substrate interaction. Previously, we have shown that Dfm1’s conserved rhomboid motifs WR and GxxxG are required for retrotranslocation (Neal et al., 2018). We utilized two mutants from our previous studies, Dfm1-AA and the Dfm1-AxxxA, in which the conserved residues in the WR or the GxxxG motif were mutated to alanine. Both Dfm1-WR/AA and Dfm1-GxxxG/AxxxA were employed in the substrate binding assay in cells expressing Hmg2, Pdr5*, or Ste6* (Figures 4F, S2A, and S2B). As a control for specificity, we tested Der1 binding to all three membrane substrates, which are not its substrate, and found no detectable association. Binding of Dfm1-AxxxA to Hmg2, Pdr5*, or Ste6* was clearly detectable and to the same extent found for binding to wild-type Dfm1. In contrast, there was no association of Dfm1-WR/AA to membrane substrates (Figures 4F, S2A, and S2B). These results indicated that the WR, and not GxxxG, motif is required for binding to ER membrane substrates tested.

### Dfm1 L1 retrotranslocation-deficient mutants are unable to bind ER membrane substrates

**Figure 5.**
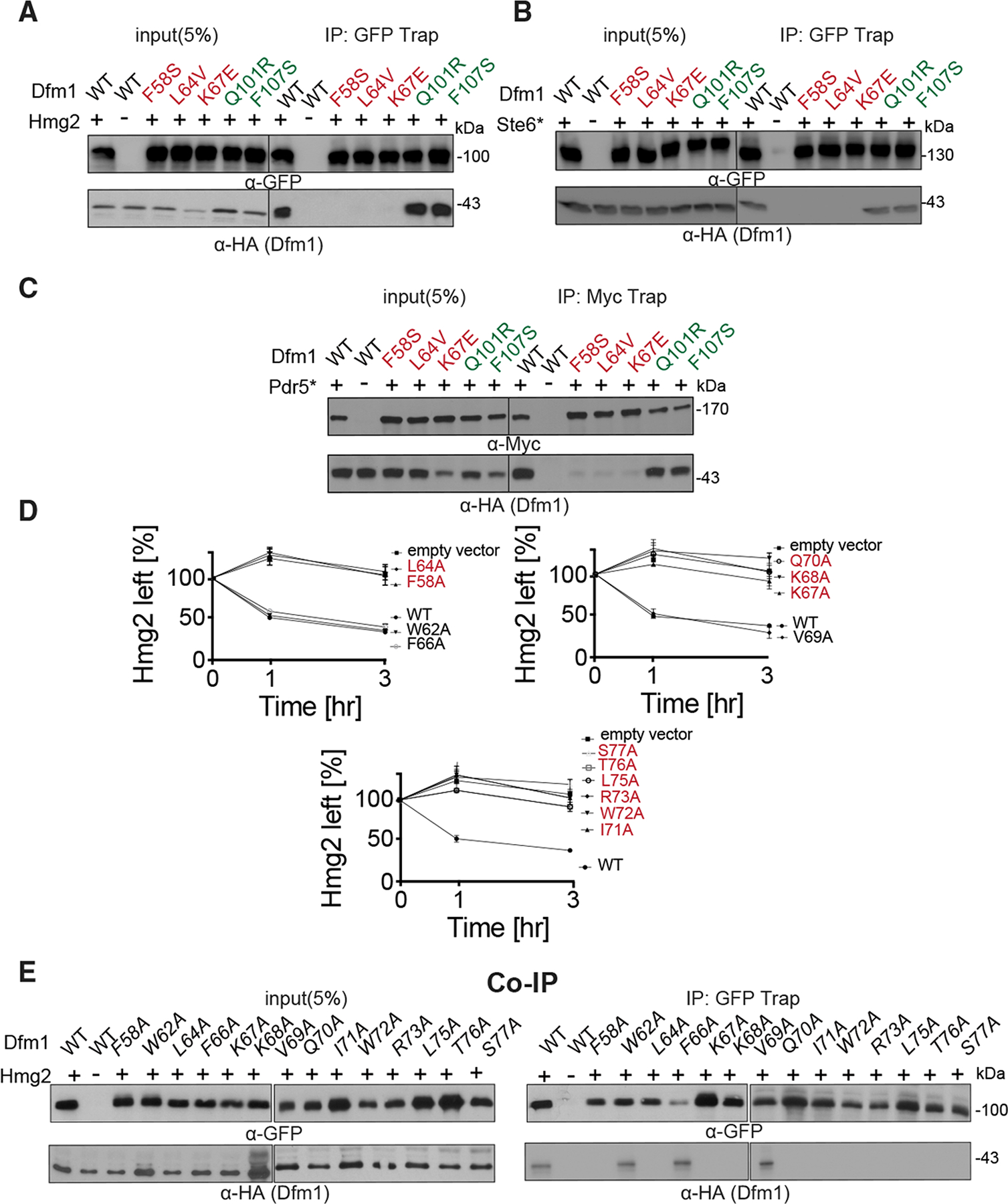
Dfm1 L1 residues are required for binding to integral membrane substrates (A) Hmg2-GFP binding to retrotranslocation-deficient Dfm1 mutants was analyzed by coIP. As negative control, cells not expressing Hmg2-GFP were used. (B) Same as (A), except Ste6* binding to retrotranslocation-deficient Dfm1 mutants was analyzed. (C) Same as (A), except Pdr5* binding to retrotranslocation-deficient Dfm1 mutants was analyzed. (D) Ala mutant scanning unveils additional L1 Dfm1 mutants required for ERAD. Strains were grown to log phase and subjected to CHX-chase. Hmg2-GFP levels were analyzed at the indicated times using flow cytometry. Histograms of 10,000 cells are shown, with the number of cells versus GFP fluorescence. Data from each time point are represented as mean ± SEM from n = 3 biological replicates. (E) Same as (A), except Hmg2-GFP binding to L1 Dfm1 mutants generated by Ala mutant scanning were analyzed by coIP.

The above results indicate the presence of additional residues along Dfm1’s transmembrane region that is involved with substrate binding. From our sequence analysis, we have identified Dfm1 L1 and L2/TM2 mutants that affect ERAD and retrotranslocation of a variety of ER membrane substrates. We next examined whether the L1 and TM2 mutants affect membrane substrate detection. We employed the substrate binding assay to analyze the association of Dfm1 mutants with various membrane substrates: Hmg2-GFP, Pdr5*-Myc, and Ste6*-GFP (Figures 5A–5C). Each substrate was immunoprecipitated with GFP Trap (for Hmg2 and Ste6*) and Myc Trap (for Pdr5*) followed by SDS-PAGE and immunoblotted for Dfm1 with α-HA, Hmg2, and Ste6* with α-GFP and Pdr5* with α-Myc. In all cases, membrane substrates were still associated with Dfm1 TM2 mutants to an extent similar to binding to wild-type Dfm1. In contrast, there was no detectable association of membrane substrates with Dfm1 L1 mutants, implying that all three L1 residues are required for membrane substrate binding.

This result suggests a critical role for L1 in membrane substrate binding, which is in agreement with previous reports that the L1 region in GlpG, a bacterial rhomboid, plays a critical role in substrate engagement (Zoll et al., 2014). Although sequence similarities across the rhomboid superfamily are low, there are highly conserved residues that cluster in the L1 region (Figure S1A). Based on GlpG’s crystal structure, L1 is embedded in the lipid bilayer, a feature that is uncommon among membrane proteins (Wang et al., 2006; Lemieux et al., 2007). The uniqueness of this motif prompted us to investigate L1 in more detail. Accordingly, we performed mutagenesis on the L1 region in which each residue was mutated to alanine. To rule out the possibility that mutants resulted in no expression or mis-localization of Dfm1, we performed western blotting on Dfm1 levels (Figure S3A). Notably, mutations within a hydrophobic patch of L1 (P55A, W56A, Y57A, I59A, Y60A, and V61A) resulted in no expression of Dfm1 (Figures S3A, S3C, and S3D). As expected, the only mutant within this cluster, which was expressed at similar levels to wild-type, was F58A; the mutant recovered and isolated from our mutagenesis screen. Based on lack of expression, it appears this hydrophobic patch plays a critical role in the structural stability for Dfm1 (Figure S3D). Furthermore, Dfm1 mutants N63A, T65A, and L74A resulted in no expression, and hence these mutants were also excluded for further functional analyses. All other mutants showing robust Dfm1 expression and exhibiting correct localization (Figure S3B) were analyzed for their effect on the steady-state levels of the self-ubiquitinating substrate SUS-GFP and ERAD of Hmg2-GFP (Figures 5D and S3C). As measured by flow cytometry, F58A, L64A, K67A, K68A, Q70A, I71A, W72A, R73A, and L75A resulted in high steady-state levels of SUS-GFP and a strong block in Hmg2-GFP degradation with GFP levels that were comparable with control cells lacking Dfm1. Notably, these same residues resulted in the inability to bind to a membrane substrate, Hmg2 (Figure 5E). In contrast, W62A, F66A, and V69A mutants did not disrupt Dfm1-mediated degradation of SUS and Hmg2 and were able to support substrate binding. Overall, mutants disrupting Dfm1’s action mapped to sites that are highly conserved in the L1 region among the rhomboid superfamily, further validating a critical role for L1 in substrate binding.

### Dynamic interaction of Dfm1 and the lipid bilayer

**Figure 6.**
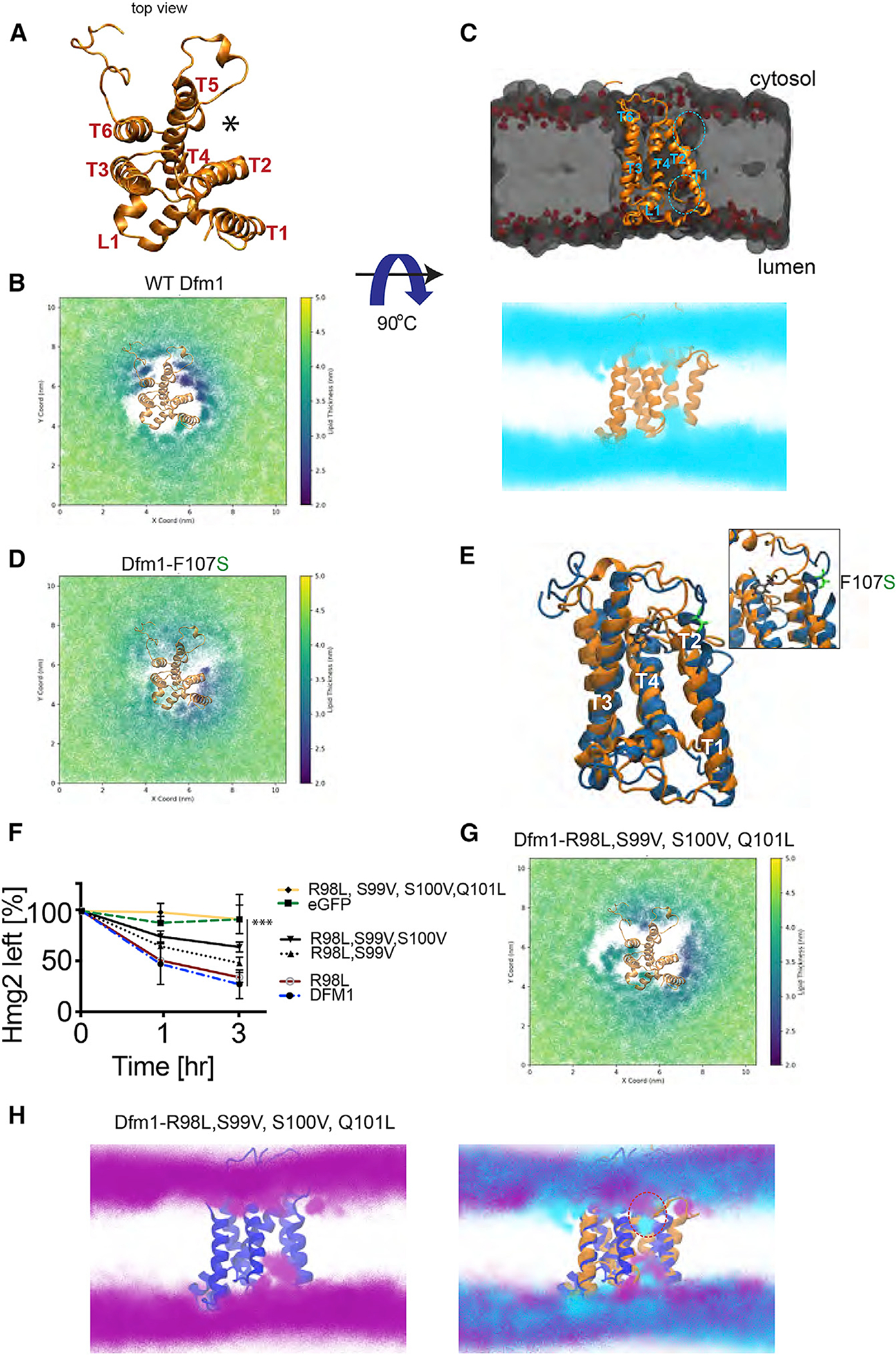
Membrane thinning by Dfm1 (A) Top view of *S. cerevisiae* derlin, Dfm1, homology model shown in gold ribbon. (B) Membrane thickness of the cytosolic leaflet is shown as x and y 2D maps of the positions of the lipid head groups every 1 ns of simulation and colored by the membrane thickness. Total thickness, i.e., the distance calculated between the upper and lower surfaces used for the analyses, is shown color coded in the 2.0 to 5.0 nm range. (C) Midpoint cross section of the membrane where Dfm1 is embedded. Dfm1 is shown in gold ribbon, the lipids are shown in a gray volumetric representation, and the phospholipid headgroup is shown in red. Lipid headgroup densities from the simulation are shown in cyan from a lateral view of TM1, TM2, and TM5. (D) Same as (B), except membrane thickness was measured for Dfm1-F107S. (E) Protein structure clusters with the highest prevalence (~50% of simulation time) of the WT protein (gold) and F107S protein (blue), highlighting the residue positional difference in F107 (black) and F107S (green). (F) CHX-chase assay was performed on *dfm1*Δ strains expressing the indicated Dfm1 mutants and Hmg2-GFP levels were measured by flow cytometry. Data are represented as mean ± SEM from n = 3 biological replicates, ***p < 0.001, repeated-measures ANOVA. (G) Same as (B), except membrane thickness was measured for Dfm1-R98L, S99V, S100V, and Q101L. (H) Same as (C), showing the lipid head group densities for the Dfm1 quad mutant (purple) with the protein structure (blue) and overlayed with the WT Dfm1 lipids (cyan) and protein (orange).

Recent work demonstrated that Dfm1’s homolog, Der1, forms a half channel with E3 ligase Hrd1 to induce lipid thinning, which facilitates in the retrotranslocation of luminal ERAD-L substrates (Wu et al., 2020). We hypothesize Dfm1 has retained membrane perturbation properties to aid in the removal of multi-spanning membrane substrates. To examine this, we first built a homology model of Dfm1, using the recently solved structure of its homolog, Der1, as a template structure (Wu et al., 2020). MD simulations were performed to examine the lipid interactions of Dfm1 embedded in a mixed lipid bilayer representative of the ER membrane. Lipid thickness in distant regions from Dfm1 was approximately 4.0–4.5 nm, which is expected for phospholipid bilayers (Bondar, 2020). In contrast, we observed rearrangement of lipids in the vicinity of Dfm1, between TM2 and TM5 (Figures 6A, 6B, and S4C). Lipids were perturbed on both the luminal (lower leaflet) and the cytoplasmic side (upper leaflet), and lipid thinning was observed with a membrane thickness of approximately 2.0–2.5 nm (Figures 6A–6C, circled in blue; Figure S4C). Furthermore, through the duration of the simulation, local lipid thinning remained in the same region, in between TM2 and TM5 (Video S1). Local lipid thinning of this magnitude has also been reported to occur in the same region of Dfm1’s paralog, Der1 (Wu et al., 2020). Interestingly, the region of membrane thinning (between Dfm1’s TM2 and TM5) is localized in an area that is known to be the lateral gate for bacterial and yeast rhomboid, GlpG and Der1, respectively (Figure 6A, indicated with asterisk) (Wang et al., 2006; Lemieux et al., 2007; Wu et al., 2020)

### Dfm1 TM2 mutants disrupt lipid thinning activity

The observed lipid deformation near TM2 and TM5 indicates an important role of these transmembrane helices in lipid thinning and retrotranslocation. We have isolated a mutant from our random mutagenesis screen, TM2 residue F107S, which is near the site of lipid perturbation (Figures 6C and 6D). This mutation has been validated in our assays for disrupting membrane substrate retrotranslocation, but not disrupting substrate binding (Figures 3A–3C and 5A–5C). To investigate how this mutant affects lipid thinning and retrotranslocation, MD simulations of the F107S mutant were performed and compared with those of the wild-type. Notably, simulations with the F107S mutation ablated lipid thinning in the vicinity of TM2 and TM5. Wild-type Dfm1 had a more consistent thinning effect around TM2 and TM5, where the membrane was around 2.0–2.5 nm, whereas F107S Dfm1 had reduced thinning in the cytosolic leaflet at approximately 3.0–3.5 nm with no effect on the ER lumen leaflet of the lipid bilayer (Figures 6D and S4C). A closer look at F107 shows this amino acid may participate in interactions with the adjacent alpha helix, TM3, implicating a structural role for F107. Indeed, F107S mutant significantly altered the structure of Dfm1, and simulations of the mutant showed an increase in the solvent-accessible surface area (SASA) of the protein (Figure S4D). Accordingly, F107 appears to play a structural role, and mutation of this led to the destabilization of Dfm1’s structure, ultimately affecting its lipid perturbation properties (Figure 6E).

Dfm1’s isoform, Der1, contains hydrophilic stretches at TM2 (residues **NH**L**ST** in bold) that are critical for lipid thinning via their interactions with the phosphate head group of the lipid bilayer (Wu et al., 2020). Dfm1 contains an analogous cluster of hydrophilic residues (RSSQ) (Figure S1A, underlined in green). Interestingly, Q101R, the retrotranslocation-deficient Dfm1 mutant isolated from our random mutagenesis screen, is within this hydrophilic cluster (RSS**Q**). To test the functional importance of the hydrophilic TM2 residues, we mutated these residues to hydrophobic amino acids to increase the hydrophobicity of TM2. The single, double, triple, and quadruple mutants were localized correctly in the microsome fraction (Figure S4E), and each mutant reduced the degradation rate of Hmg2, with the strongest stabilization observed with the quadruple mutant (R98L, S99V, S100V, Q101L) (Figure 6F). We next investigated how these mutants disrupt lipid thinning through MD simulations with the quadruple mutant (R98L, S99V, S100V, Q101L) Dfm1. Like the F107S mutation, the quadruple TM2 mutant affected lipid thinning on the cytosolic leaflet in the vicinity of TM2 and TM5, where the membrane thickness was increased to approximately 4.0–4.5 nm (Figures 6G, 6H, and S4C). Although Dfm1 is mainly embedded within the lipid bilayer, its surface has prominent charge clusters that allow for strong electrostatic interactions with lipid headgroups in membranes. Notably, the RSSQ hydrophilic cluster is localized within a positive charge pocket, where, in our simulations, a phosphate headgroup was found to bind strongly (Figures 6C and S4F). This pocket seems to be critical for a significant lipid perturbation in the TM2 and TM5 lateral gate. When the RSSQ cluster was mutated to LVVL, the pocket became neutral/slightly negatively charged, and the phosphate binding motif was lost (Figures 6H and S4F). Furthermore, the quad mutant destabilized Dfm1’s tertiary structure, and an alteration in TM5 and TM6’s angle resulted in a significantly more open structure (Figure S4F). Altogether, our analyses implicate that the hydrophilic cluster on TMD2 is poised to induce lipid thinning directly by strongly interacting with the phosphate head groups of the lipid membrane.

### Derlin-1 homologous mutants disrupt ERAD of CFTR

The studies above have identified sequence features of yeast derlin Dfm1, which are important for its retrotranslocation function via membrane substrate detection and lipid thinning. The closest human homolog to Dfm1 is Derlin-1, the most well-characterized human derlin to date (Sun et al., 2006; Greenblatt et al., 2012; Suzuki et al., 2012; You et al., 2017). In fact, Dfm1 has higher sequence similarity to human Derlin-1 than to its yeast paralog Der1 (Sato and Hampton, 2006). Like Dfm1, Derlin-1 possesses a Shp tail for recruiting p97/Cdc48, along with the conserved WR and GxxxG motifs, which have been shown to be critical for its retrotranslocation function (Greenblatt et al., 2011). We wanted to perform a similar random mutagenesis screen on full-length human Derlin-1 by leveraging our established screen in *S. cerevisiae*. We first verified whether full-length human DERLIN-1 or its other ERAD-participating paralog, DERLIN-2 gene, can complement the *dfm1*Δ phenotype in *S. cerevisiae*. DERLIN-1 or DERLIN-2 yeast-optimized coding sequence was inserted into the yeast expression plasmid and transformed into *dfm1*Δ cells expressing ERAD-M substrate, SUS-GFP. Both Derlin-1 and Derlin-2 steady-state levels were detectable by western blotting (Figure S5A). However, both Derlin-1 and Derlin-2 were not able to degrade Hmg2-GFP, implicating that both human derlins are not able to functionally complement yeast derlin Dfm1 (Figure S5B).

**Figure 7.**
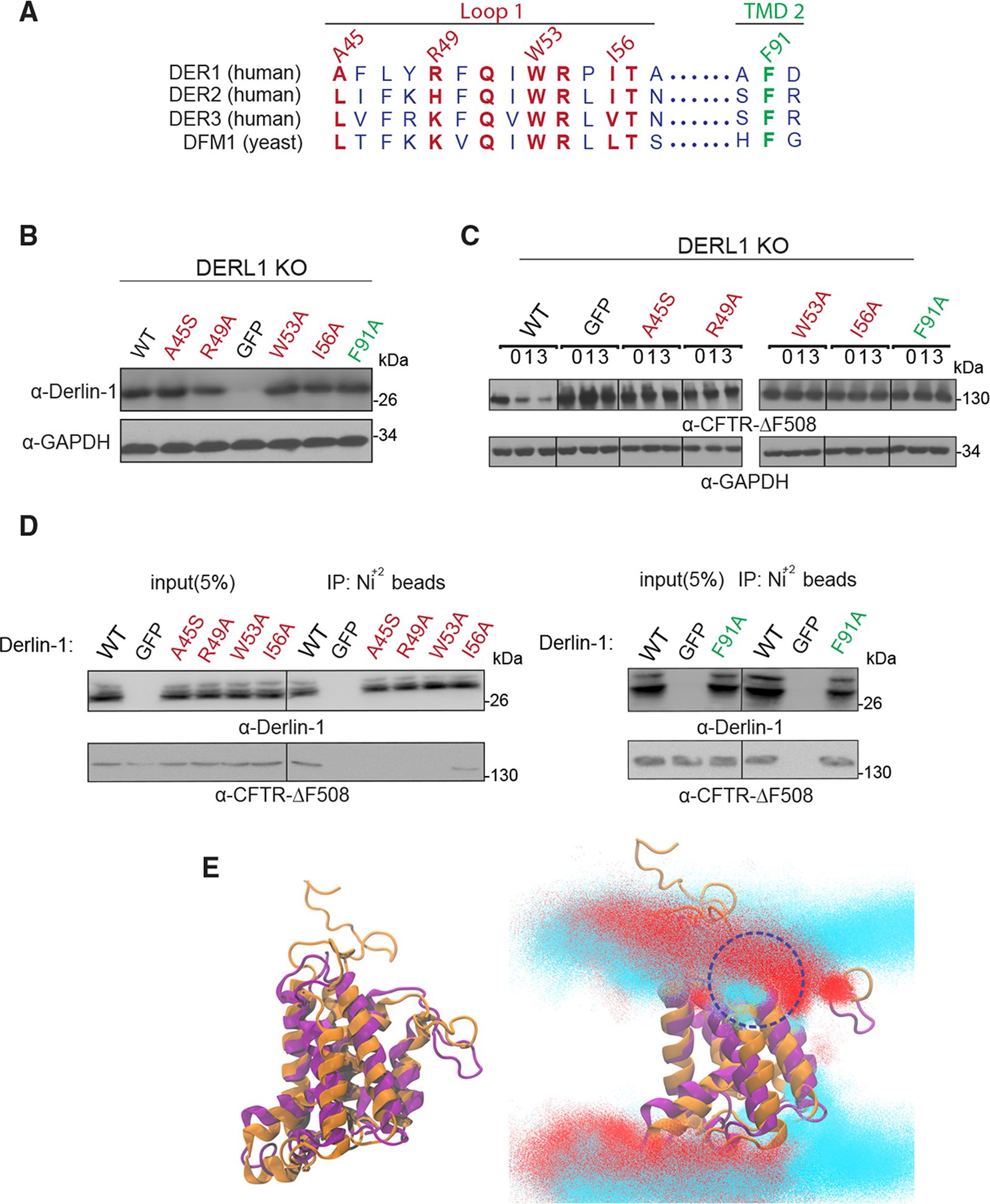
Conservation of human derlin, Derlin-1, and ERAD function (A) Alignment of *H. sapiens* Derlin-1, 2, and 3 and *S. cerevisiae* Dfm1. Similarly or identically conserved residues in L1 and TM2 are highlighted in red and green, respectively. (B) DERLIN-1 KO HEK293T cells were transfected with DERLIN-1 as described under STAR Methods. 50 μg of lysate was subjected to immunoblotting for Derlin-1 with α-Derlin-1 and GAPDH with α-GAPDH. (C) Cycloheximide-chase were performed in DERLIN-1 KO HEK293T cells co-transfected with DERLIN-1 and ΔF508-CFTR. 48 h after co-transfection, cells were treated with 100 μg/mL CHX and harvested at the indicated chase times for immunoblotting of CFTR. (D) HEK293T lysates were incubated with Ni^+2^ beads, and the bound proteins were eluted with SDS-PAGE sample buffer, resolved by SDS-PAGE, and analyzed by immunoblotting with specific antibodies against Derlin-1 and CFTR. (E) Protein structure clusters with the highest prevalence (~50% of simulation time) of WT Dfm1 protein (gold) and human Derlin-1 (blue). Lipid head group densities from simulations of human Derlin-1 (red) with the protein structure (purple) and overlayed with the WT Dfm1 lipids (cyan) and protein structure (orange).

We next utilized the mammalian system to examine sequence requirements for human derlins’ function. Interestingly, a subset of Dfm1 residues that were identified from random mutagenesis and Ala mutant scanning (L1: L64, K68, L75 and TM2: F107S) are similarly conserved in its human homolog, Derlin-1 (L1: A45, R49, I56, and TM2: F91, respectively) (Figure 7A). We determined whether these conserved Derlin-1 residues with similar properties are critical for ERAD. We performed site-directed mutagenesis on Derlin-1’s similarly conserved residues that were critical for yeast Dfm1’s actions. As a control, we generated the W53A mutant in the WR motif, because this motif has been shown to be required for human Derlin-1 retrotranslocation function (Greenblatt et al., 2011). All Derlin-1 mutants displayed robust expression and correct localization to the ER (Figures 7B and S5C). A well-characterized multi-spanning membrane substrate for Derlin-1 is the clinically important disease-causing mutant cystic fibrosis transmembrane conductance regulator (CFTR)-ΔF508 (Sun et al., 2006). We have successfully generated a Derlin-1 knockout cell line expressing CFTRΔF508. Using this cell line, effects of Derlin-1 mutants were directly tested with CHX-chase assays of CFTRΔF508 stability. Remarkably, in all cases, the normally degraded CFTRΔF508 was stabilized by all Derlin-1 mutants and levels similar to GFP only and WR mutant control (Figure 7C). We next examined whether the Derlin-1 L1 and TM2 mutants affected their binding to CFTRΔF508. Each Derlin-1 mutant was subjected to pull-down experiments with Ni^2+^ agarose beads. A fraction of CFTRΔF508 (~5%) copurified with wild-type Derlin-1 (Figure 7D). As a control for specificity, CFTRΔF508 did not bind to resins in GFP-only cells. CFTRΔF508 associated with Derlin-1 TM2 mutant F91A to an extent similar to wild-type Derlin-1. In contrast, there was no detectable association of CFTRΔF508 with Derlin-1 mutants (A45S, R49A, W53A) and decreased association of CFTRΔF508 (~1%) with Derlin-1 mutant I56A, implying that Derlin-1 L1 residues also contribute to membrane substrate binding of CFTRΔF508 (Figure 7D).

Subsequently, we next determined whether yeast Dfm1’s lipid thinning function is also conserved in human Derlin-1. We generated a homology model of human Derlin-1 and notably, the structure was similar to yeast Dfm1 (Figure 6E). Next, MD simulations were performed with Derlin-1 embedded in a mixed lipid bilayer representative of the ER membrane. Similar to yeast Dfm1, we observed lipid thinning between TM2 and TM5. In particular, TM2 strongly interacted with phosphate head groups in the upper leaflet of the lipid bilayer (Figure 7E, circled in blue). Overall, these results indicate yeast Dfm1 mechanistic substrate engagement and lipid thinning actions can be generalized and extended to human derlin function.

Recent cryoelectron microscopy (cryo-EM) work demonstrated that Derlin-1 forms a tetrameric channel, and this complex was predicted to allow a single-transmembrane helix to pass through its pore during retrotranslocation (Rao et al., 2021). We wanted to determine whether Derlin-1 was able to induce lipid thinning as a tetrameric complex. To examine this, we performed MD simulations to examine the lipid interactions of the Derlin-1 tetramer embedded in a mixed lipid bilayer representative of the ER membrane. Lipid thickness in distant regions from the complex was approximately 4.0–4.5 nm, which is expected for phospholipid bilayers (Bondar, 2020). Whereas monomeric Derlin-1 displayed significant lipid thinning near TM2 and TM5, we observed minor rearrangement of lipids in the periphery of the Derlin-1 tetramer (near TM1 and TM6) with membrane thinning ranging from 3.0 to 4.0 nm, implicating lipid distortion of this magnitude may be sufficient in facilitating membrane protein retrotranslocation (Figure S5D). These results indicate yeast Dfm1 mechanistic substrate engagement and lipid thinning actions can be generalized and extended to human derlin function.

## DISCUSSION

In this study, we unveiled the mechanistic features of derlins, a subclass of rhomboid-like proteins that are widely represented in ERAD. We discovered L1 and TM2 residues are critical for the function of both yeast Dfm1 and human Derlin-1. Closer analysis reveals that L1 mutants are defective in detecting membrane substrates, suggesting the L1 regions are required for substrate binding as previously shown for rhomboid proteases (Zoll et al., 2014). Our studies also provide the first evidence that derlin rhomboid pseudoproteases have retained membrane-perturbing properties. Specifically, we observed a cluster of hydrophilic residues in TM2 of Dfm1 that directly mediate lipid thinning in the juxtaposition membrane. Our studies demonstrate that derlins utilize the unique properties of the rhomboid superfamily for carrying out the widely conserved and critical process of membrane protein retrotranslocation.

Our coimmunoprecipitation (coIP) experiments with Dfm1 mutants and chimeras indicate substrate detection requires Dfm1’s SHP tail through Cdc48 recruitment. Notably, Cdc48, which is recruited by Dfm1, directly binds to the polyubiquitin chain of substrates. This was evident through treatment with a deubiquitinase or excess polyubiquitin chains, which disrupted binding of ubiquitinated substrate, Hmg2, to the Dfm1-Cdc48 complex. In addition, several members of the rhomboid superfamily recruit Cdc48/p97 to associate directly with the polyubiquitin chains attached to their substrates. For example, human rhomboid protease Rhbdl4 and *S. pombe* rhomboid pseudoprotease, Rbd2, recruit Cdc48/p97 through their (VCP-binding motif) VBM and (Src homology region 2 domain-containing phosphatase) SHP motifs, respectively, for direct interaction of polyubiquitin chains on substrates (Fleig et al., 2012; Hwang et al., 2016). Thus, binding to polyubiquitin chains of substrates appears to be a feature utilized by a subset of the rhomboid superfamily.

We show that Dfm1 L1 region mediates the recognition of integral membrane substrates. An important question that arises from our observations is: how does L1 gain substrate access? One possibility is that Dfm1’s L1 region is poised to attract membrane substrates possessing unstable and/or positively charged transmembrane helices. For example, membrane substrates with helix-breaking residues and limited hydrophobicity move readily into the hydrophilic interior cavity of GlpG prior to cleavage (Moin and Urban, 2012). Additionally, the mammalian rhomboid protease Rhbdl4 selectively binds substrates with positively charged transmembrane helices (Fleig et al., 2012). Indeed, MD simulation of Dfm1 with H_2_O molecules demonstrated that the interior of Dfm1 is hydrophilic, implicating this property is leveraged to lure in membrane substrates with hydrophilic exposed residues or positively charged transmembrane helices (Figure S4A). Interestingly, a similar simulation with Der1 demonstrated one side of the protein was hydrophilic, supporting its role in functioning as a “half channel” for transporting soluble membrane substrates (Figure S4B). Alternatively, L1 can aid in the diffusion of Dfm1 through the lipid bilayer to survey for membrane substrates destined for retrotranslocation (Kreutzberger et al., 2019). This alternative mode is supported by a previous study suggesting a subset of the rhomboid family diffuses rapidly through the lipid bilayer to survey for membrane substrates. Nevertheless, L1 client recruitment and binding function appears to be an evolutionarily conserved process as previously demonstrated for Dfm1’s bacteria homolog, GlpG, which employs L1 substrate interactions for optimal alignment of substrate’s backbone to the proteolytic site (Zoll et al., 2014). Overall, based on the requirement of Dfm1 L1 and Cdc48 recruitment for substrate association, we suggest a model of at least two coordinated functions of Dfm1 in substrate detection. We propose a model in which the Dfm1 L1 region brings the substrate in close proximity to Dfm1, allowing for concomitant binding to the polyubiquitin chain attached to the substrate.

Cells must have a strategy in place for overcoming the thermodynamic barrier of removing hydrophobic integral membrane proteins from their stable home within the lipid bilayer (Marinko et al., 2019). For example, the magnitude of this energetic barrier has been shown by bacteriorhodopsin, a membrane protein, which exhibits a free energy difference of 230 ± 40 kcal/mol between its native and unfolded state (Müller et al., 2002). Derlins’ lipid thinning function can meet this high-energy demand by reducing the lipid permeability barrier to allow ease of substrate movement across the membrane (Neal et al., 2018; Wu et al., 2020). Notably, Dfm1’s rhomboid predecessors are believed to bind to their substrates within the membrane to partially (and passively) unfold the TM helices of substrates prior to proteolytic cleavage (Wang et al., 2007; Moin and Urban, 2012). Because rhomboid pseudoproteases, such as Dfm1 and Der1, have retained the overall architecture of rhomboid proteases, they may also bind and unwind substrates, further lowering the energetic barrier for substrate removal.

Derlin-1 has been implicated in several diseases, such as viral infection, cancer, cystic fibrosis, and neurological dysfunctions (Kandel and Neal, 2020). Accordingly, determining the mechanistic features associated with human derlin function is critical for understanding its vast roles in normal physiology and pathology. Interestingly, Derlin-1 requires the widely conserved rhomboid motifs, WR and GxxxG, for retrotranslocation (Greenblatt et al., 2011). We observed that retrotranslocation-deficient Dfm1 mutants are located at sites that are conserved in human derlin Derlin-1. Site-directed mutagenesis of Derlin-1 at these conserved sites ablates substrate detection and retrotranslocation of its multi-spanning substrate, CFTR-ΔF508. A recent cryo-EM study demonstrates that Derlin-1 forms a tetrameric channel (Rao et al., 2021). The authors proposed a mechanism of a single transmembrane helix traversing through the Derlin-1 channel during retrotranslocation. Notably, TM2-TM5 lines the inner channel of the tetramer complex. Based on our simulations with yeast Dfm1, this is the region where the lipid bilayer is distorted significantly. However, simulations with tetrameric Derlin-1 showed a minor rearrangement of lipids in the periphery of the Derlin-1 complex with membrane thickness ranging from 3.0 to 4.0 nm (Figure S5D). Nevertheless, significant lipid thinning between TM2 and TM5 was observed with monomeric Derlin-1 (Figure 7E). It is possible that Derlin-1 exists in different oligomeric states. For instance, Derlin-1 has been found to be in a complex with several other ERAD components, such has HRD1, SEL1, and VCP. Furthermore, a previous study demonstrated that the monomeric form of Derlin-1 is sufficient in mediating ERAD, and there is a pool of inactive Derlin-1 homodimer complex that is modulated in response to ER stress (Crawshaw et al., 2007). In agreement with this, rhomboid proteins from diverse species function as monomers (Kreutzberger and Urban, 2018; Škerle et al., 2020). In these cases, lipid thinning through TM2-TM5 would be critical for monomeric Derlin-1 function. Furthermore, a closer look at the Derlin-1 tetramer model showed that the TM1-L1-TM3 gate lined the outer channel of the core and is aligned with our data for making the first contact with incoming membrane substrates. The authors predicted many hydrophobic residues within L1 (including Derlin-1 I56 residue we characterized in this study for substrate binding) are required for substrate engagement through this gate. This suggests an intriguing model in which derlin-mediated retrotranslocation of membrane substrates utilize both rhomboid features and channel activity. A high-resolution structure of derlins with their respective substrate along with crosslinking experiments to map out how substrates bind to Derlin-1 would be invaluable for investigating this phenomenon in the future.

Our study has sought out to understand the mechanisms associated with the widely critical function of multi-spanning membrane substrate retrotranslocation. A recent structure of human Derlin-1 demonstrates it forms an oligomeric complex, which is conducive to having channel activity. Similarly, Der1, along with E3 ligase Hrd1, form a channel to function in ERAD-L retrotranslocation. These findings implicate that derlin rhomboid pseudoproteases may employ both channel activity and its rhomboid features through L1-mediated substrate binding and lipid thinning to aid in the dislocation of membrane and luminal substrates, respectively. Overall, this study provides functional insights of derlin rhomboid pseudoproteases, which will ultimately aid in the therapeutic design against these rhomboid-like proteins that are associated with a plethora of maladies, including cancer, cystic fibrosis, and neurological dysfunctions.

## STAR★METHODS

### RESOURCE AVAILABILITY

#### Lead contact

Further information and requests for resources and reagents should be directed to and will be fulfilled by the Lead Contact, Sonya Neal (seneal@ucsd.edu).

#### Materials availability

Plasmids and yeast strains generated in this study is available from our laboratory.

#### Data and code availability

Original/source data for figures is available upon request.

This paper does not report original code.

Any additional information required to reanalyze the data reported in this work paper is available from the Lead Contact upon request

### EXPERIMENTAL MODEL AND SUBJECT DETAILS

#### Microbe strains

*E. coli* DH5 alpha, S. cerevisae BY4747 and S288C

See Table S2 for complete list of yeast strains and their corresponding genotypes.

#### Cell lines

##### HEK293T cells (ATCC)

Authentication testing was performed on established human cell lines regardless of the application, and testing was done, at minimum, at the beginning and end of experimental work. For HEK293T human cell lines, short tandem repeat (STR) profiling was performed and compared to results from online databases of human cell line STR profiles (ANSI/ATCC ASN-0002-2011 Authentication of Human Cell Lines: Standardization of STR Profiling. ANSI eStandard Store.)

### METHOD DETAILS

#### Yeast and Bacteria Growth Media

Standard yeast *Saccharomyces cerevisiae* growth media were used as previously described (Hampton and Rine, 1994), including yeast extract-peptone-dextrose (YPD) medium and ammonia-based synthetic complete dextrose (SC) and ammonia-based synthetic minimal dextrose (SD) medium supplemented with 2% dextrose and amino acids to enable growth of auxotrophic strains at 30°C. *Escherichia coli* Top10 cells were grown in standard LB media with ampicillin at 37°C as previously described (Gardner et al., 1998). HEK293 cells were cultured in DMEM medium supplemented with 10% FBS.

#### Plasmids and Strains

Plasmids used in this study are listed in Table S1. Plasmids for this work were generated using standard molecular biological techniques (Sato et al., 2009) and verified by sequencing (Eton Bioscience, Inc.). Primer information is available upon request. Full-length human DERLIN-1 cDNA was obtained by G-block synthesis (Eton Bioscience, Inc.) and subcloned into pcDNA3.1/Myc-His(+)A (Invitrogen) to express Derlin-1 with the myc epitope at the C terminus. The KHN (pRH1958) and KWW (pRH1960) plasmids were a gift from Davis Ng (National University of Singapore, Singapore). The Ste6* plasmid (pRH2058) was a gift from S. Michaelis (Johns Hopkins School of Medicine, MD). The Pdr5* plasmid was a gift from Dieter H. Wolf (University of Stuttgart, Stuttgart, Germany). The pcDNA3.1-ΔF508-CFTR plasmid was a gift from J. Brodsky (University of Pittsburgh, PA).

A complete list of yeast strains and their corresponding genotypes are listed in Table S2. All strains used in this work were derived from S288C or Resgen. Yeast strains were transformed with DNA or PCR fragments using the standard LiOAc method (Ito et al., 1983). Null alleles were generated by using PCR to amplify a selection marker flanked by 50 base pairs of the 5′ and 3′ regions, which are immediately adjacent to the coding region of the gene to be deleted. The selectable markers used for making null alleles were genes encoding resistance to G418 or CloNat/nourseothricin. After transformation, strains with drug markers were plated onto YPD followed by replica-plating onto YPD plates containing (500 μg/mL G418 or 200 μg/mL nourseothricin). All gene deletions were confirmed by PCR.

#### dfm1Δ strain handling

Due to rapid suppression nature of *dfm1*Δ null strains, freshly transformed *dfm1*Δ null cells with the respective ERAD-M substrates should be used in every assay. Generation of Dfm1 mutant strains and troubleshooting guidelines are found in (Bhaduri and Neal, 2021).

#### Homology modeling

To build the 3D model of yeast derlin, Dfm1 protein on the template of yeast derlin Der1, the Phyre2 system was utilized(Kelley et al., 2015). Initially, the primary sequence is scanned against a database of 10 million known sequences for homologs via PSI-Blast. From here, homologous sequences are organized into an evolutionary fingerprint through Hidden Markov Models. Evolutionary fingerprints and Hidden Markov Models are made for the 65,000 known 3D structures to create a database of known structures. A scan of the evolutionary fingerprint with the database creates an alignment to known structures ranked by confidence of homology. This alignment generates a 3D threaded model with excellent accuracy even when sequence identity is less than 15%, and in addition is able to reliably detect extremely remote homology (Kelley et al., 2015).

#### Molecular dynamics simulation

The Dfm1 protein structure used in the MD simulations was built through homology modeling with the SWISS-MODEL structure prediction server (Waterhouse et al., 2018). Der1 was the primary homologous structure for the predicted model of Dfm1 and the generated model covered residues 31–236 of Dfm1. Systems were prepared for MD using CHARMM-GUI’s membrane builder to place the protein in a 100Å × 100Å lipid patch and to apply any necessary amino acid mutations (Jo et al., 2008). The lipid composition was built to be representative of the ER membrane with a number percent composition of 47% POPC [1-palmitoyl-2-oleoyl-glycero-3-phosphocholine], 20% POPE [1-palmitoyl-2-oleoyl-sn-glycero-3-phosphoethanolamine], 15% cholesterol, 11% POPI [1-palmitoyl-2-oleoyl-sn-glycero-3-phosphoinositol], and 7% POPS [1-palmitoyl-2-oleoyl-sn-glycero-3-phospho-L-serine] (van Meer et al., 2008; Casares, Escribá and Rosselló, 2019). The system was solvated with the TIP3P water model and included 0.15M NaCl salt concentration. MD simulations were run with the CHARMM36m forcefield in an NPT ensemble at 310K and 1.01325 bar using the GROMACS 2018.3 MD engine (Abraham et al., 2015; Huang et al., 2017). Systems were energy minimized and subsequently equilibrated in a stepwise manner, slowly relaxing the restraints, for a total of 2 ns, with the first 100ns of zero restraints simulation being considered as additional equilibration time. Both the wild-type protein and the F107S mutant were simulated in triplicate with each replicate running for ~700ns. A quad mutant Dfm1(Figures 6 and S4), WT Der1 (PDB 6VJZ) (Figure S4), and tetrameric human Der1 (PDB 7CZB) (Figure 7) were simulated for ~400ns in triplicate under the same conditions (Wu et al., 2020; Rao et al., 2021).

#### MD analyses

Membrane thickness calculations were performed using MDTraj to first split the lipids into separate leaflets and then calculate the distances between lipid headgroups of opposing leaflets for each frame of the simulation (McGibbon et al., 2015). Membrane thickness was defined to be an average of the 3 shortest trans-leaflet distances between headgroups for each lipid. Protein structures were clustered using GROMACS’ cluster command which conducted GROMOS clustering on the backbone atoms of the protein structure (Daura et al., 1999). SASA calculations were performed using the SASA command in GROMACS with a probe radius of 1.4Å. Visualizations were rendered using VMD and POVRay3.0 (Humphrey, Dalke and Schulten, 1996). Protein surface charge was determined through Adaptive Poisson-Boltzmann Solver (APBS) electrostatics calculations (Jurrus et al., 2018).

#### Random mutagenesis of Dfm1

pRH2013 plasmid containing DFM1 driven from its native promoter was amplified by PCR using a high fidelity Phusion polymerase (control) and error prone Mutazyme 2 to introduce point mutations into DFM1. Specifically, 500ng of template DNA (pRH2013) and 20 cycles of PCR were used to obtain an average of 1–3 point mutations within DFM1, excluding the genetic region encoding the SHP motifs as per protocol instructions. Mutagenized DFM1 was amplified using high fidelity Phusion polymerase and treated with Dpn1 at 37°C overnight to digest the original unmutagenized template followed by PCR cleanup of mutagenized DFM1 using Promega wizard PCR cleanup kit. In parallel, backbone plasmid from pRH2013 was prepared by overnight digestion with Spe1 and PshA1 and then purified from 0.8% agarose gel. For homologous recombination of mutagenized DFM1 with pRH2013 backbone, linearized pRH2013 and purified mutagenized DFM1 were co-transformed into *dfm1*Δ*hrd1*Δ yeast cells containing TDH3pr-SUS-GFP using a 1:9 backbone to insert ratio. Recombinants were selected on SC-Leu and incubated at 30°C. Resulting transformants were selected for high colony fluorescence, indicating their inability to degrade the optical retrotranslocation reporter, SUS-GFP. Plasmids were recovered from selected yeast transformants and transformed into *E. coli*. Plasmids were recovered using Promega Wizard Plus SV Miniprep kit, as per manufacturer’s protocol and sent to ETON for sequencing using T7 (forward) and T3 (reverse) universal primers. Results for sequencing were aligned to wild-type DFM1 and mutated regions were identified using in house python scripts. Mutants containing one point mutation and no early stop codons verified by both forward and reverse strands were selected as mutants of interest.

#### Plasmid recovery from transformants

Plasmid extractions was performed as described in Flagg et al. (2019). Transformants were inoculated into 3-ml YPD and grown overnight. The following day, 1 mL of YPD was added to stationary phase cultures, which were then allowed to grow for an hour at 30°. The entire culture was then pelleted and resuspended in 250 μL of resuspension buffer from a Promega Wizard Plus SV Miniprep kit (A1460). Resuspended cells were lysed with beads for 5 min in a multi-vortexer. Lysed cells and supernatant were then collected by nesting the microcentrifuge tube into a 15-ml conical tube, piercing the 2-ml microcentrifuge tube with a needle, and spinning the nested tubes at 2,000 rpm for 2 min. Lysed cells and cell lysate were then thoroughly resuspended, and the remainder of the miniprep was carried out according to the manufacturer’s protocol.

#### Cell culture, transfections and immunoblotting

Both wild-type and DERLIN-1 knockout HEK293T cell lines (ATCC) (kindly provided by Dr. Hideki Nishitoh, University of Miyazaki) were cultured in Dulbecco’s modified Eagle medium (25 mM glucose, sodium pyruvate) (Invitrogen) and grown at 37°C and 5% CO_2_. The media was supplemented with 10% fetal bovine serum (FBS) (Atlanta Biological). Both pcDNA3.1-DERLIN-1 and pcDNA3.1-ΔF508-CFTR were co-transfected into HEK293T cells in a 1:1 ratio using Lipofectamine® LTX (Invitrogen) according to manufacturer’s instructions. Forty-eight hours after transfection, cells were lysed in lysis buffer (50 mM HEPES pH 7.5, 150 mM NaCl, 1% NP40, 0.1% SDS and 0.5% sodium deoxycholate, and 1 mM EDTA supplemented with protease inhibitors for 1 hour on ice. After a centrifugation at 21,000 × *g* for 10 min at 4°C, the protein concentration was measured using the BCA protein assay kit (Pierce) and resuspended in SDS sample buffer and subjected to immunoblotting analysis. Equal amounts of protein extracts (30 μg) were separated by SDS-PAGE, transferred on nitrocellulose membrane and immunoblotted for anti-GAPDH (BioRad), anti-Derlin-1 (ABclonal, Inc.) and anti-CFTR (CFTR Antibodies Distribution Program).

#### *In Vivo* Retrotranslocation Assay

*in vivo* retrotranslocation assay was performed as described in Neal et al., (2019). Cells in log phase (OD_600_ 0.2–0.3) were treated with MG132 (benzyloxycarbonyl-Leu-Leu-aldehyde, Sigma) at a final concentration of 25 μg/mL (25 mg/mL stock dissolved in DMSO) for 2 hours at 30°C and GGPP (Geranylgeranyl pyrophosphate ammonium salt, Sigma) at a final concentration of 11 μM for 1 hour at 30°C and 15 ODs of cells were pelleted. Cells were resuspended in H_2_0, centrifuged and lysed with the addition of 0.5 mM glass beads and 400 μL of XL buffer (1.2 M sorbitol, 5 mM EDTA, 0.1 M KH_2_PO_4_, final pH 7.5) with PIs, followed by vortexing in 1minute intervals for 6–8 min at 4°C. Lysates were combined and clarified by centrifugation at 2,500 g for 5 min. Clarified lysate was ultracentrifuged at 100,000 g for 15 min to separate pellet (P100) and supernatant fraction (S100). P100 pellet was resuspended in 200 μL SUME (1% SDS, 8 M Urea, 10 mM MOPS, pH 6.8, 10 mM EDTA) with PIs and 5 mM N-ethyl maleimide (NEM, Sigma) followed by addition of 600 μL immunoprecipitation buffer (IPB) with PIs and NEM. S100 supernatant was added directly to IPB with PIs and NEM. 15 μL of rabbit polyclonal anti-GFP antisera (C. Zuker, University of California, San Diego) was added to P100 and S100 fractions for immunoprecipitation (IP) of Hmg2-GFP. Samples were incubated on ice for 5 minutes, clarified at 14,000 g for 5 min and removed to a new eppendorf tube and incubated overnight at 4°C. 100 μL of equilibrated Protein A-Sepharose in IPB (50% w/v) (Amersham Biosciences) was added and incubated for 2 h at 4°C. Proteins A beads were washed twice with IPB and washed once more with IP wash buffer (50 mM NaCl, 10 mM Tris), aspirated to dryness, resuspended in 2x Urea sample buffer (8 M urea, 4% SDS, 1mM DTT, 125 mM Tris, pH 6.8), and incubated at 55°C for 10 min. IPs were resolved by 8% SDS-PAGE, transferred to nitrocellulose, and immunoblotted with monoclonal anti-ubiquitin (Fred Hutchinson Cancer Center, Seattle) and anti-GFP (Clontech, Mountain View, CA). Goat anti-mouse (Jackson ImmunoResearch, West Grove, PA) and goat anti-rabbit (Bio-Rad) conjugated with horseradish peroxidase (HRP) recognized the primary antibodies. Western Lightning® Plus (Perkin Elmer, Watham, MA) chemiluminescence reagents were used for immunodetection.

#### Cycloheximide-Chase Assay

For yeast cells, cycloheximide chase assays were performed as previously described (Sato et al., 2009). Cells were grown to log-phase (OD_600_ 0.2–.03) and cycloheximide was added to a final concentration of 50 μg/mL. At each time point, a constant volume of culture was removed and lysed. Lysis was initiated with addition of 100 μL SUME with PIs and glass beads, followed by vortexing for 4 min. 100 μL of 2xUSB was added followed by incubation at 55°C for 10 min. Samples were clarified by centrifugation and analyzed by SDS-PAGE and immunoblotting.

For HEK293T, cells were transfected with the indicated plasmids and 48 h later, cells were digested with trypsin and passaged in fresh medium supplemented with cycloheximide (20 μg/mL) for the indicated times. Equivalent volume of cell suspensions was harvested at different time points. Cell extracts were subjected to immunoblotting analysis as described above.

#### Cdc48 Microsome Association Assay

Yeast strains were grown to log phase (OD_600_ 0.2–0.3) and 15 ODs of cells were pelleted. Cells were resuspended in H_2_0, centrifuged and lysed with the addition of 0.5 mM glass beads and 400 μL of XL buffer with PIs and vortexed in 1-minute intervals for 6–8 min at 4°C. Lysates were combined and clarified by centrifugation at 2,500 g *for 5 min. 50 μL* of lysate was transferred to another tube and designated as total fraction (T). The rest of clarified lysate was centrifuged at 20,000 × g for 5 min to separate microsome pellet (P) and cytosolic supernatant fraction (S). An equivalent volume of 2xUSB was added to T, P and S fractions followed by solubilization at 55°C for 10 min. Samples were clarified by centrifugation, analyzed by SDS-PAGE and immunoblotted for Cdc48 and PGK1 with α-CDC48 (1:5,000) and α-PGK1(1:5,000) respectively.

#### Native Co-IP

Cultures from various yeast strains were grown to OD_600_ 0.2–.45 and 15 ODs of cells were pelleted, rinsed with H_2_0 and lysed with 0.5 mM glass beads in 400 μL of MF buffer supplemented with protease inhibitors. This was followed by vortexing at 1-minute intervals for 6–8 minutes at 4°C. Lysates were combined and clarified by centrifugation at 2,500 g for 5 min followed by centrifugation at 14,000 g for 15 min to obtain the microsomal pellet. The microsomal pellet was resuspended in 1 mL of Tween IP buffer (500 mM NaCl, 50 mM Tris, pH 7.5, 10 mM EDTA. 1.5% Tween-20) and incubated on ice for 30 minutes. Lysates were then centrifuged for 30 min at 14,000 × g, and the supernatant was incubated overnight with 10 μL of equilibrated GFP-Trap® agarose (ChromoTek Inc., Hauppauge, NY) at 4°C. The next day, the GFP-Trap® agarose beads were combined to one tube, washed once with non-detergent IP buffer, washed once more with IP wash buffer and resuspended in 100 μL of 2xUSB. Samples were resolved on 8% SDS-PAGE and immunoblotted for ubiquitin with anti-Ub, Cdc48 with α-CDC48, Hmg2-GFP with α-GFP, Dfm1-HA with α-HA, and Ste6*-GFP with α-GFP.

HEK293 cells were treated with 50 μM MG132 for 12 hours and lysed by douncing in minimal buffer (10 mM HEPES pH 8.0, 1 mM MgCl2, 2 mM KCl supplemented with protease inhibitor cocktails, and centrifuged at 35,000 rpm for 30 min at 4°C. Microsomal pellet was resuspended in solubilization buffer (50 mM HEPES pH 8.0, 300 mM NaCl, 10 mM imidizole, 1 mM MgCl_2_, 2 mM of KCl, 1% digitonin, and protease inhibitors) followed by incubation for 3 hours at 4°C. Lysates were centrifuged at 80,000 rpm for 30 min at 4°C followed by incubation of supernatant with Ni^+2^ beads at 4°C overnight. The next day, beads were washed twice with solubilization buffer, and incubated in elution buffer (solubilization buffer + 300 mM imidazole) to elute Derlin from Ni^+2^ beads, and resuspended in SDS sample buffer for immunoblotting analysis.

#### *In Vivo* Cross-linking

15 OD of cells from indicated strains were lysed with zymolyase and treated with vehicle (DMSO) or DSP (dithiobis(succinimidyl propionate) for 40 min. Microsomes were isolated and solubilized in Tween IP buffer supplemented with protease inhibitors and Hmg2-GFP was immunoprecipitated with GFP-Trap® agarose beads.

#### Free Polyubiquitin Competition Test

The ability of polyubiquitin to compete for Cdc48 binding to ubiquitinated Hmg2-GFP was adapted from Co-IP of Hmg2-GFP as described above. Microsomes were prepared and resuspended in 1 mL of Tween IP buffer supplemented with protease inhibitors followed by incubation on ice for 20 minutes. Lysates were then centrifuged for 30 min at 14,000 × g, and the supernatant was incubated with (2, 5, 10 or 20 μg) Lys48-linked polyubiquitin chains (BostonBiochem®) or buffer for one hour at 4°C. 30 μL of equilibrated GFP-Trap® agarose was added to each tube, and they were nutated overnight at 4°C. The next day, the GFP-Trap® agarose beads were washed with Tween IP buffer, washed once more with IP wash buffer and resuspended in 100 μL of 2xUSB. Samples were resolved on 8% SDS-PAGE and immunoblotted for Cdc48 with α-CDC48, Hmg2-GFP with α-GFP, and Dfm1-HA with α-HA.

#### Proteolytic Removal of Ubiquitin from Hmg2-GFP

Ubiquitin removal was accomplished with the broadly active Usp2 ubiquitin protease as previously described (Garza et al., 2009), except that human recombinant Usp2Core (LifeSensors Inc., Malvern, PA) was used, and leupeptin and NEM were excluded from all buffers. Briefly, microsome fraction solubilized in 1 mL of Tween buffer was was incubated with 10 μL of Usp2Core (5 μg) for 1 hr at 37°C. The reaction was quenched with 200 *μl of* SUME with PIs and retrotranslocated Hmg2-GFP was immunoprecipitated as described above. 20 μL of IP was used for detection of Hmg2-GFP with anti-GFP. 30 μL of equilibrated GFP-Trap® agarose was added and the sample was nutated overnight at 4°C. The next day, the GFP-Trap® agarose beads were washed with Tween IP buffer, washed once more with IP wash buffer and resuspended in 100 μL of 2xUSB. Samples were resolved on 8% SDS-PAGE and immunoblotted for ubiquitin with anti-Ub, Cdc48 with α-CDC48, Hmg2-GFP with α-GFP, and Dfm1-HA with α-HA.

#### Immunofluorescent staining

HEK293T cells were cultured on confocal slides, transfected with plasmids to express wild-type and Derlin-1 mutants. 36 hours later, cells were fixed with 2% paraformaldehyde at room temperature for 10 minutes, permeabilized with 0.1% Triton X-100 in PBS, washed three times in 0.5% bovine serum albumin and 0.15% glycine at (pH 7.4) in phosphate-buffered saline, reacted with anti-His antibody (ABclonal), and incubated with Alexa 568 conjugated anti-mouse secondary antibody (ThermoFisher). After washing with 0.5% bovine serum albumin and 0.15% glycine at (pH 7.4) in phosphate-buffered saline, the coverslips were mounted for confocal microscopy.

### QUANTIFICATION AND STATISTICAL ANALYSIS

ImageJ (NIH) was used for all western blot quantifications. Band intensities were measured directly from films scanned in high resolution (600 dpi) in TIF file format. “Mean gray value” was set for band intensity measurements. In such experiments, a representative western blot was shown and band intensities were normalized to PGK1 loading control and quantified. t = 0 was taken as 100% and data is represented as mean ± SEM from at least three experiments. GraphPad Prism was used for statistical analysis. Nested t test, unpaired t test or one-way factorial ANOVA followed by Bonferroni’s post hoc analysis were applied to compare data. Significance was indicated as follow: n.s, not significant; * p < 0.05, ** p < 0.01, *** p < 0.001, **** p < 0.0001. The investigators were blinded during data analysis.

## Supplementary Material

1

2

3

## Figures and Tables

**Table:** KEY RESOURCES TABLE

REAGENT or RESOURCE	SOURCE	IDENTIFIER
Antibodies		
Mouse monoclonal anti-GFP	Clontech Laboratories, Inc.	Cat#632381; RRID: AB_2313808
Mouse monoclonal anti-HA	Thermo Fisher Scientific	Cat#32-6700; RRID: AB_2533092
Rabbit polyclonal anti-Myc	Genscript	Cat#A00172; RRID: AB_914457
Rabbit polyclonal anti-Cdc48	Neal et al., 2018	N/A
Mouse monoclonal anti-PGK	Thermo Fisher Scientific	Cat#459250; RRID: AB_2569747
Mouse monoclonal anti-Ubiquitin	Richard Gardner: University of Washington	N/A
Rabbit polyclonal anti-Derlin-1	Abclonal	A8508; RRID: AB_2769151
Mouse anti-GAPDH	BIO-RAD	Cat#MCA4740; RRID:AB_2107457
Rabbit anti-CFTR	Antibodies Distribution Program	N/A
Bacterial and virus strains		
*Escherichia coli* Top10 Competent Cells	ThermoFisher Scientific	Cat#C404010
Chemicals, peptides, and recombinant proteins		
MG132 (benzyloxycarbonyl-Leu-Leu-aldehyde)	Sigma-Aldrich	474787; CAS: 133407-82-6
Cycloheximide	Sigma-Aldrich	C7698; CAS: 66-819
Protein A Sepharose	GE Healthcare	17-0780-01
Usp2core	LifeSensors	DB501
AQUApure Tetra-Ub Chains (K48-linked)	R&D Systems	UC-210B
DpnI restriction enzyme	New England Biolabs	R0176L
High Fidelity Phusion polymerase	New England Biolabs	M0530L
PCR Clean-Up System	Promega	A9282
Ampicillin	Biopioneer	C0029
Nourseothricin	Neta Scientific, Inc	RPI-N51200-1.0
G418	Biopioneer	C0050
Lipofectamine® LTX (Invitrogen)	ThermoFisher Scientific	A12621
GFP-Trap agarose	ChromoTek	gta-20
Myc-Trap agarose	ChromoTek	yta-20
Geranylgeranyl pyrophosphate ammonium salt	Millipore Sigma	G6025
3,3-Dithio- *bis*-(sulfosuccinimidyl) propionate (DSP)	Millipore Sigma	322133
Protein A-Sepharose	Millipore Sigma	GE17-0780-01
DIOC6 (3,3′-Dihexyloxacarbocyanine Iodide)	ThermoFisher Scientific	D273
Critical commercial assays		
GeneMorph II Random Mutagenesis Kit	Agilent Technologies	200550
Experimental models: Cell lines		
Human: HEK293 cell line		ATCC
Experimental models: Organisms/strains		
*Saccharomyces cerevisiae* BY4741	GE Dharmacon	Cat#YSC1048
*Saccharomyces cerevisiae* S288C	This study	N/A
Additional yeast strains used: refer to Table S2	This study	
Recombinant DNA		
Plasmids used: refer to Table S1	This study	N/A
pRABBIT IgG IRES-EmGFP Positive Control Vecto	ThermoFisher Scientific	A39243
Software and algorithms		
Prism 7 for Mac	GraphPad Software	https://www.graphpad.com/scientific-software/prism/
ImageJ	NIH	https://imagej.nih.gov/ij/
FlowJo	Vashistha et al., 2016	https://www.fiowjo.com/solutions/fiowjo
BD Accuri C6	BD Accuri	Cat #653122
PyMOL	Schrodinger,LLC	https://pymol.org/2/
Protein Data Bank	RCSB PDB	https://www.rcsb.org/

## Highlights

- Dfm1 selectively binds ERAD-targeted membrane substrates
- Polyubiquitin chains bind directly to Cdc48 recruited by Dfm1
- Derlin lipid thinning facilitates removal of integral membrane substrates in the ER
- Substrate engagement and lipid thinning are conserved derlin features
