# Supplementary material for: Derlin rhomboid pseudoproteases employ substrate engagement and lipid distortion to enable the retrotranslocation of ERAD membrane substrates
Source: Cell Rep. Author manuscript; Available in PMC 2021 Dec 3. (PMC8641752; doi:10.1016/j.celrep.2021.109840)
Supplement: 1 [file NIHMS1749865-supplement-1.pdf]

Cell Reports, Volume *37*

Supplemental information

Derlin rhomboid pseudoproteases employ substrate engagement and lipid distortion to enable the retrotranslocation of ERAD membrane substrates

Anahita Nejatfard, Nicholas Wauer, Satarupa Bhaduri, Adam Conn, Saroj Gourkanti, Narinderbir Singh, Tiffany Kuo, Rachel Kandel, Rommie E. Amaro, and Sonya E. Neal

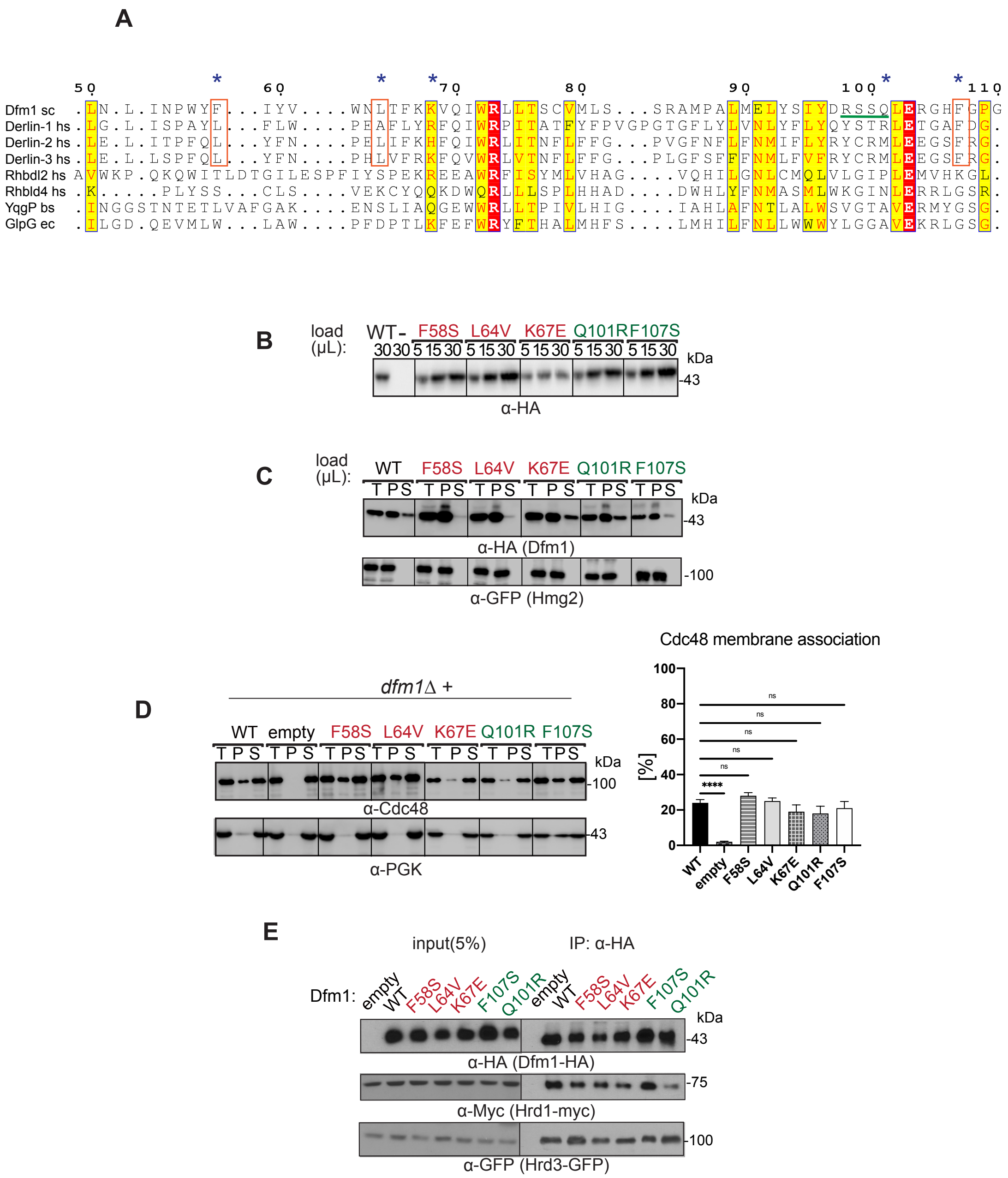
A
Dfm1 sc
Derlin-1 hs
Derlin-2 hs
Derlin-3 hs
Rhbdl2 hs
Rhbld4 hs
YqgP bs
GlpG ec
B
load (µL):
WT
-
F58S
L64V
K67E
Q101R
F107S
kDa
-43
α-HA
C
load (µL):
WT
F58S
L64V
K67E
Q101R
F107S
T P S
kDa
-43
α-HA (Dfm1)
-100
α-GFP (Hmg2)
D
dfm1Δ +
WT
empty
F58S
L64V
K67E
Q101R
F107S
T P S
kDa
-100
α-Cdc48
-43
α-PGK
Cdc48 membrane association
[%]
ns
****
WT
empty
F58S
L64V
K67E
Q101R
F107S
E
input(5%)
IP: α-HA
Dfm1:
empty
WT
F58S
L64V
K67E
F107S
Q101R
kDa
-43
α-HA (Dfm1-HA)
-75
α-Myc (Hrd1-myc)
-100
α-GFP (Hrd3-GFP)

Figure S1. Dfm1 is a rhomboid pseudoprotease. Related to Figures 1 & 2. **(A)** TCoffee alignment of the transmembrane regions of Derlin-1, Derlin-2 and Derlin-3, Rhbdl2 and Rhbdl4 from H. sapiens; Dfm1 from S. cerevisiae; YqgP from B. subtilis; and Glpg from E. coli. Identically and similarly conserved residues are highlighted in red and yellow respectively. Also, residues selected from loss of function screen is indicated by blue asterisks. **(B)** Dfm1 mutants are stably expressed. Stability of Dfm1 mutants were measured by loading increasing amounts of lysates (5 μL, 15 μL, and 30 μL) on SDS-PAGE followed by immunoblotting with α-HA. **(C)** Dfm1 mutants localize to the ER. Total cell lysate (T) from the indicated strains were separated into soluble cytosolic fraction (S) and pellet microsomal fraction (P) upon centrifugation at 14,000 x g. Each fraction was analyzed by SDS-PAGE and immunoblotted for Dfm1 mutants with α-HA and ER-localized Hmg2 with α-GFP. **(D)** Dfm1 mutants do not disrupt its Cdc48 recruitment function. Same as (A), except Cdc48 recruitment was analyzed by immunoblotting for Cdc48 with α-Cdc48 and Pgk1 with α-Pgk1. The graph shows the quantification of Cdc48 in the pellet fractions of the respective cells as measured from ImageJ. Data is represented as percentage of Cdc48 that is bound to pellet fraction and is shown as mean ± SEM from n=3 biological replicates, **** $p < 0.0001$, Oneway ANOVA. **(E)** Association of retrotranslocation deficient mutants to E3 ligase Hrd1 and Hrd3 was analyzed by co-IP. As a negative control, cells not expressing Dfm1 were used.

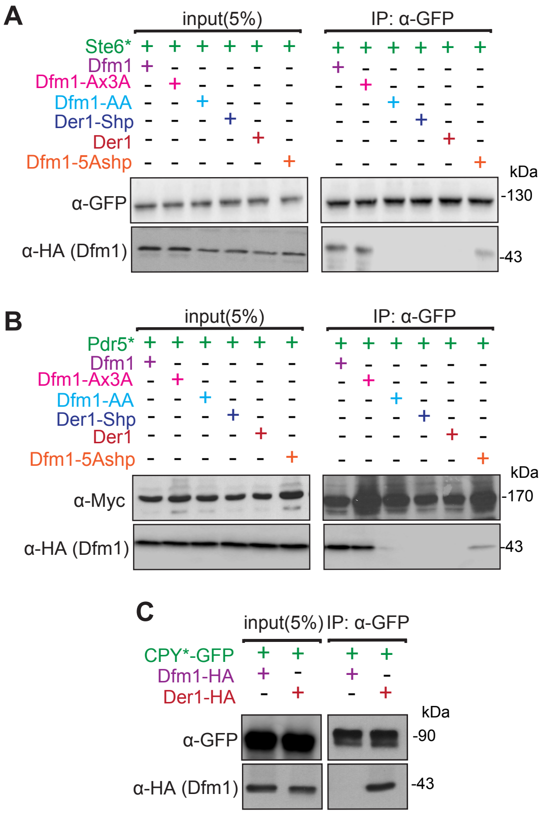
A
input(5%)
IP: α-GFP
Ste6*
Dfm1
Dfm1-Ax3A
Dfm1-AA
Der1-Shp
Der1
Dfm1-5Ashp
kDa
α-GFP
-130
α-HA (Dfm1)
-43
B
input(5%)
IP: α-GFP
Pdr5*
Dfm1
Dfm1-Ax3A
Dfm1-AA
Der1-Shp
Der1
Dfm1-5Ashp
kDa
α-Myc
-170
α-HA (Dfm1)
-43
C
input(5%)
IP: α-GFP
CPY*-GFP
Dfm1-HA
Der1-HA
kDa
α-GFP
-90
α-HA (Dfm1)
-43

Fig. S2. Dfm1 selectively binds to integral membrane substrates. Related to Figure 4. **(A)** Co-IP was used to analyzed Ste6* binding to Dfm1 variants; Dfm1-5Ashp, Dfm1-AA, and Dfm1-Ax3A. D was analyzed by co-IP As a control for specificity, cells expressing Der1-HA were used. **(B)** Same as **(A)**, except substrate Pdr5* was used in co-IP. **(C)** Same as **(A)** except substrate CPY* was used in co-IP.

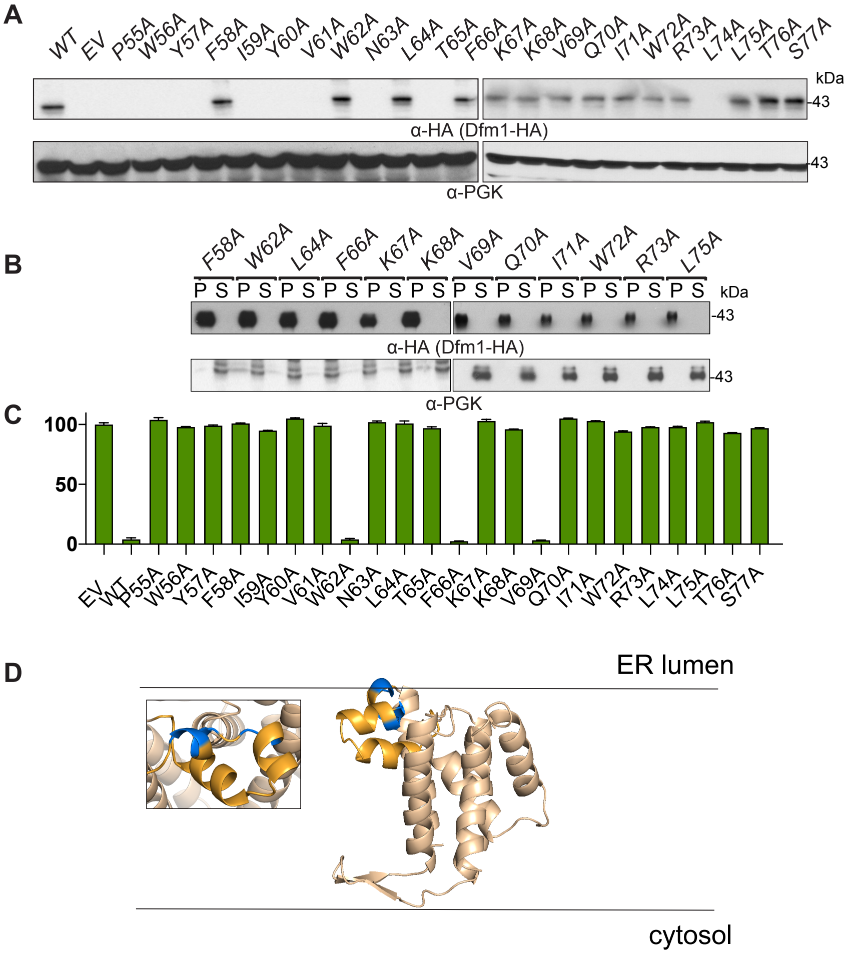

A
WT EV P55A W56A Y57A F58A I59A Y60A V61A W62A N63A L64A T65A F66A K67A K68A V69A Q70A I71A W72A R73A L74A L75A T76A S77A
kDa
-43
α-HA (Dfm1-HA)
-43
α-PGK
B
F58A W62A L64A F66A K67A K68A V69A Q70A I71A W72A R73A L75A
P S P S P S P S P S P S P S P S P S P S P S P S
kDa
-43
α-HA (Dfm1-HA)
-43
α-PGK
C
100
50
0
EV WT P55A W56A Y57A F58A I59A Y60A V61A W62A N63A L64A T65A F66A K67A K68A V69A Q70A I71A W72A R73A L74A L75A T76A S77A
D
ER lumen
cytosol

Fig S3. Expression, localization and functionality of Dfm1 Loop 1 mutants. Related to Figure 5. **(A)** Stability of Dfm1 L1 mutants generated by Ala mutant scanning were measured by loading 30 μL of lysates on SDS-PAGE followed by immunoblotting with α-HA. **(B)** The indicated strains were grown to log phase and were subjected to flow cytometry to measure steady-levels of fluorescent membrane substrate, SUS-GFP. Histograms of 10,000 cells are shown, indicating the mean FITC-A value. **(C)** Dfm1 L1 mutants localize to the ER. The indicated strains were separated into soluble cytosolic fraction (S) and pellet microsomal fraction (P) upon centrifugation at 14,000 x g. Each fraction was analyzed by SDS-PAGE and immunoblotted for Dfm1 mutants with α-HA and cytosol-localized Pgk with α-Pgk. **(D)** Homology model of Dfm1. Position of non-polar residues (hydrophobic patch) are indicated in blue.

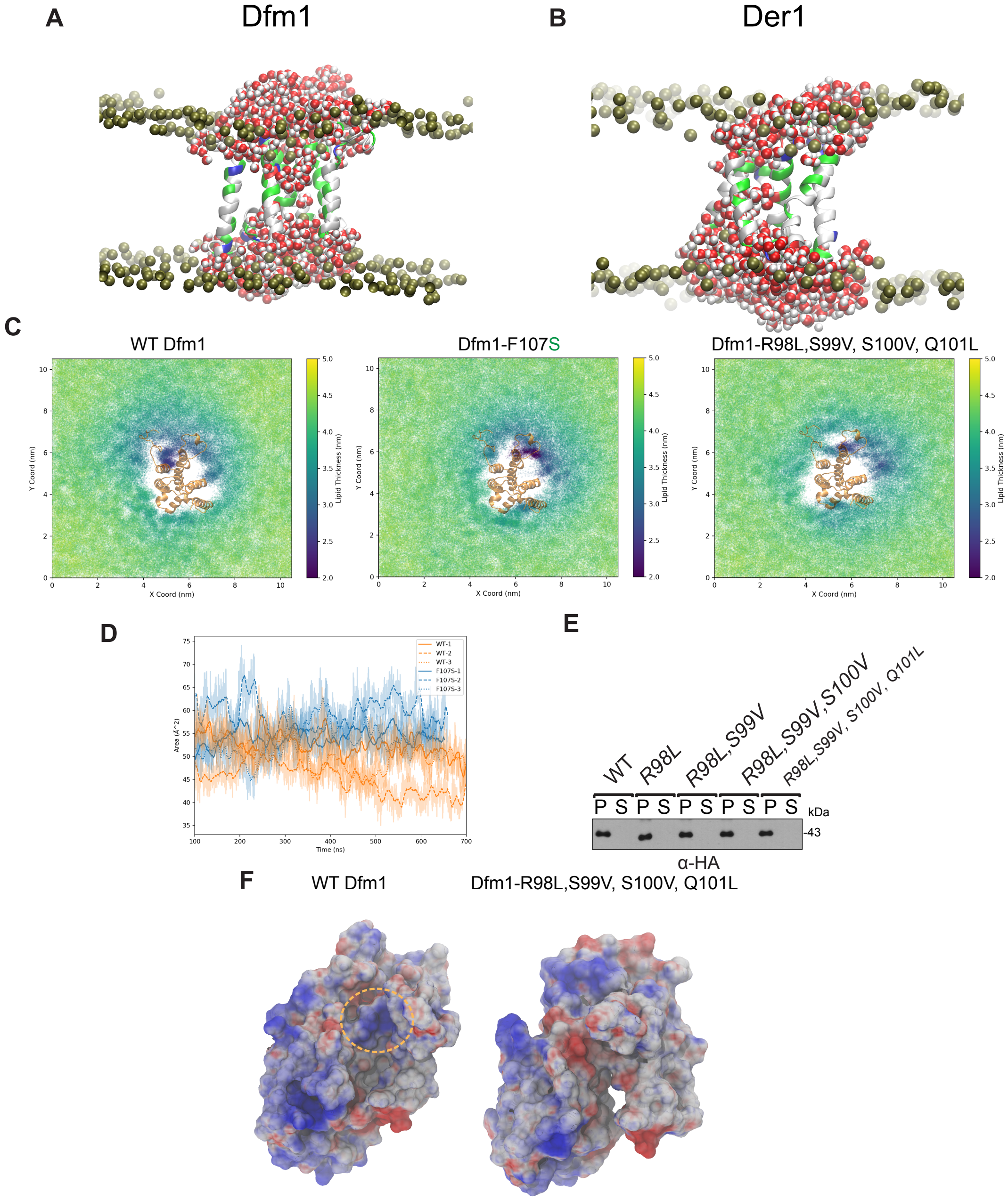
A
Dfm1
B
Der1
C
WT Dfm1
Dfm1-F107S
Dfm1-R98L,S99V, S100V, Q101L
Y Coord (nm)
X Coord (nm)
Lipid Thickness (nm)
D
Area (Å^2)
Time (ns)
WT-1
WT-2
WT-3
F107S-1
F107S-2
F107S-3
E
WT
R98L
R98L, S99V
R98L, S99V, S100V
R98L, S99V, S100V, Q101L
P S P S P S P S P S
kDa
-43
α-HA
F
WT Dfm1
Dfm1-R98L,S99V, S100V, Q101L

Fig. S4. Dfm1's TMD2 is required for lipid thinning. Related to Figure 5. **(A)** Simulation of S. cerevisiae derlin, Dfm1 homology model embedded in a mixed lipid bilayer (lipid composition in methods). Dfm1 is shown in multicolored ribbon representing the residue type (white is hydrophobic, green is polar, blue is positive, red is negative), water molecules are shown in red and white, and the phospholipid head group is shown in gold. **(B)** Same as **(A),** except the simulation was with S. cerevisiae derlin, Der1. **(C)** Membrane thickness of the ER lumen leaflet is shown as x and y 2D maps of the positions of the lipid head groups every 1ns of simulation and colored by the membrane thickness at that timepoint/location. The Dfm1 protein model is overlayed to show the relative locations of membrane thinning by native, mutant Dfm1-F107S and quad mutant Dfm1-R98L, S99V, S100V, Q101L. Total thickness, i.e. the distance calculated between the upper and lower surfaces used for the analyses, is shown color-coded according to a 2.0 to 5.0 nm range. **(D)** SASA calculation of the solvent exposed surface of WT and F107S Dfm1. The shaded area shows the raw data while the line marks a 10 ns moving average of the data. **(E)** Dfm1 mutants localize to the ER. Indicated strains were separated into soluble cytosolic fraction (S) and pellet microsomal fraction (P) upon centrifugation at 14,000 x g. Each fraction was analyzed by SDSPAGE and immunoblotted for Dfm1 mutants with anti-HA. **(F)** Protein surface showing the surface charge (positive charge is blue, negative charge is red) of both WT and quad mutant Dfm1 according to Adaptive Poisson-Boltzmann Solver (APBS) electrostatics calculations.

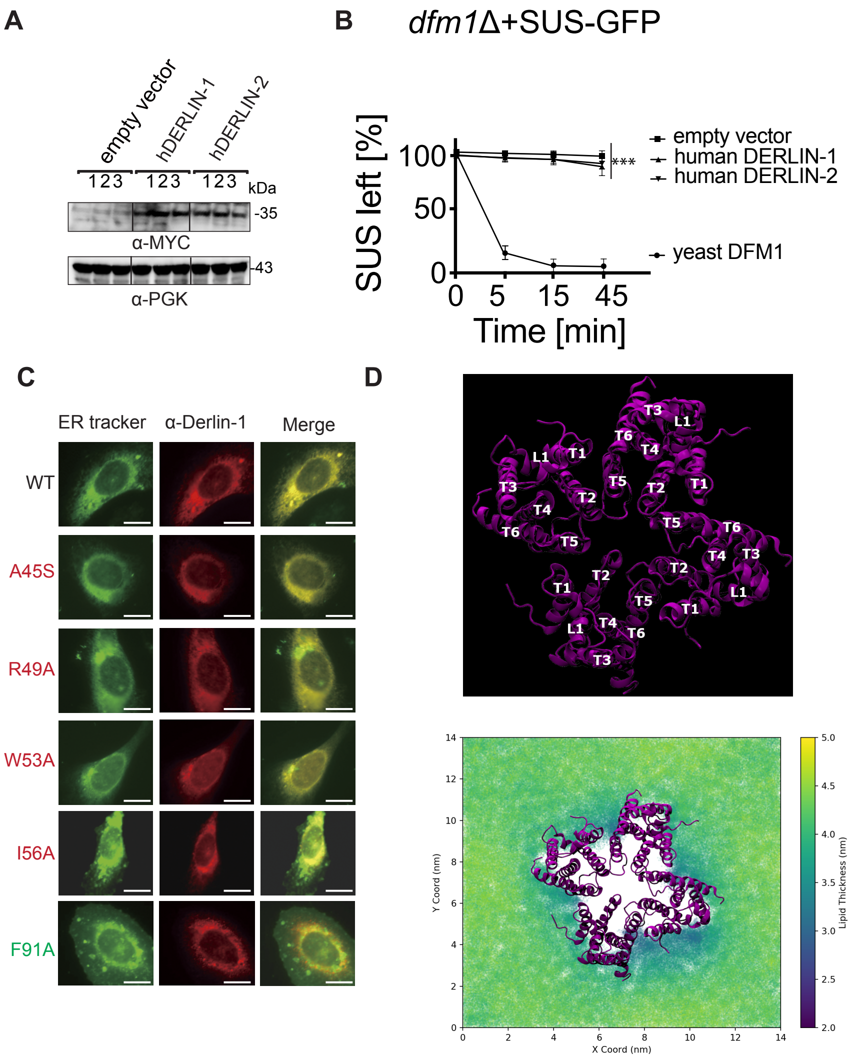

A
empty vector
hDERLIN-1
hDERLIN-2
123
123
123
kDa
-35
α-MYC
-43
α-PGK
B
dfm1Δ+SUS-GFP
SUS left [%]
100
50
0
0
5
15
45
Time [min]
***
empty vector
human DERLIN-1
human DERLIN-2
yeast DFM1
C
ER tracker
α-Derlin-1
Merge
WT
A45S
R49A
W53A
I56A
F91A
D
Y Coord (nm)
X Coord (nm)
Lipid Thickness (nm)

Fig. S5. Human Derlin-1 requires Loop 1 and TMD2 for substrate binding and lipid thinning respectively. Related to Figure 7. **(A)** Indicated strains (n=3) with heterologous expression of human Derlin-1 or Derlin-2 were grown to log-phase, lysed and analyzed by SDS-PAGE and immunoblotted for their steady-state levels by alpha-Myc. **(B)** dfm1Δ strains containing SUS-GFP along with human Derlin-1, Derlin-2 or empty vector add back were grown to log-phase and degradation was measured by CHX-chase analysis. After CHX addition, Hmg2-GFP levels were measured by flow cytometry. Data is represented as mean ± SEM from tn=3 biological replicates, $***p < 0.001$, Repeated Measures ANOVA. **(C)** HEK293T cells with indicated Derlin-1 mutants were stained with anti-MYC (Derlin-1, red) or DIOC6 (ER, green) and examined byconfocal microscopy in the mid-plane of each cell. Scale bar indicates 10 μM. **(D)** The human Derlin-1 tetrameric model is overlayed with the membrane thickness plot to show the relative locations of membrane thinning. Total thickness, i.e. the distance calculated between the upper and lower surfaces used for the analyses, is shown color-coded according to a 2.0 to 5.0 nm range.

Table S1. Plasmids used in this study, Related to Figures 1-7

Plasmid	Gene		
pSN4	YCp	LEU2	pDER1-SHP-WR
pSN55	YCp	LEU2	pDER1-SHP-GX3G
pSN58	YCp	LEU2	pDER1-SHP-WR+GX3G
pSN59	YCp	LEU2	pDFM1-F107S
pSN60	YCp	LEU2	pDFM1-L64V
pSN90	YCp	LEU2	pDFM1
pSN93	YCp	LEU2	pDFM1-K67E
pSN94	YCp	LEU2	pDFM1-Q101R
pSN95	YCp	LEU2	pDFM1-F58S
pSN204	YCp	LEU2	pDFM1-P55A
pSN205	YCp	LEU2	pDFM1-W56A
pSN206	YCp	LEU2	pDFM1-Y57A
pSN207	YCp	LEU2	pDFM1-F58A
pSN208	YCp	LEU2	pDFM1-I59A
pSN209	YCp	LEU2	pDFM1-Y60A
pSN210	YCp	LEU2	pDFM1-V61A
pSN211	YCp	LEU2	pDFM1-W62S
pSN212	YCp	LEU2	pDFM1-N63A

pSN213	YCp LEU2	pDFM1-L64A
pSN214	YCp LEU2	pDFM1-T65A
pSN215	YCp LEU2	pDFM1-F66A
pSN216	YCp LEU2	pDFM1-K67A
pSN217	YCp LEU2	pDFM1-K68A
pSN218	YCp LEU2	pDFM1-V69A
pSN219	YCp LEU2	pDFM1-Q70A
pSN220	YCp LEU2	pDFM1-I71A
pSN221	YCp LEU2	pDFM1-W72A
pSN222	YCp LEU2	pDFM1-R73A
pSN223	YCp LEU2	pDFM1-L74A
pSN224	YCp LEU2	pDFM1-L75A
pSN225	YCp LEU2	pDFM1-T76A
pSN226	YCp LEU2	pDFM1-S77A
pSN177	YCp URA3	pCPY*-GFP
pSN170	pcDNA 3.1A	pDERLIN-1
pSN171	pcDNA 3.1A	pDERLIN-1-R49A
pSN172	pcDNA 3.1A	pDERLIN-1-W53A
pSN173	pcDNA 3.1A	pDERLIN-1-F91A
pSN174	pcDNA 3.1A	pDERLIN-1-A45S

pSN175	pcDNA 3.1A	pDERLIN-1-I56A
pSN162	YCp LEU2	pDFM1-5Ashp-3HA
pSN161	YCp LEU2	pDER1-SHP-3HA
prH2826	YCp LEU2	pDFM1-AA-3HA
prH2812	YCp LEU2	pDFM1-Ax$_3$A-3HA
pSN115	YIp TRP1	pSUS-GFP
pSN178	pcDNA3.1	pCFTR
pSN179	pcDNA3.1	pCFTRΔF508
pRH613	YIp URA3/ADE2	pTDH3-HMG2-GFP
pRH2058	2 μ URA3	pPGK-STE6-166-3HA-GFP
pSN227	YCp LEU2	pDFM1-R98L
pSN228	YCp LEU2	pDFM1- R98L,S99V
pSN229	YCp LEU2	pDFM1- R98L,S99V, S100V
pSN230	YCp LEU2	pDFM1- R98L,S99V, S100V, Q101L
pSN193	YIp LEU2/ADE2	pADH1-DERLIN-1-MYC
PSN194	YIp LEU2/ADE2	pADH1-DERLIN-2-MYC

Table S2. Yeast strains used in this study, Related to Figures 1-7

Strain	Genotype	Reference
SEN 54	Mata *ade2-101 met2 lys2-801 his3Δ200 trp1::hisG leu2Δ ura3-52::URA3::TDH3pr-HMG2-GFP dfm1Δ::KanMX CEN::LEU2::prDER-SHP-WR*	This study
SEN 56	Mata *ade2-101 met2 lys2-801 his3Δ200 trp1::hisG leu2Δ ura3-52::URA3::TDH3pr-HMG2-GFP dfm1Δ::KanMX CEN::LEU2::prDER-SHP-GX3G*	This study
SEN 58	Mata *ade2-101 met2 lys2-801 his3Δ200 trp1::hisG leu2Δ ura3-52::URA3::TDH3pr-HMG2-GFP dfm1Δ::KanMX CEN::LEU2::prDER-SHP-WR+GX3G*	This study
SEN 59	Mata *ade2-101 met2 lys2-801 his3Δ200 trp1::hisG leu2Δ ura3-52::URA3::TDH3pr-HMG2-GFP dfm1Δ::KanMX CEN::LEU2::prDER-SHP*	This study
SEN 112	Mata *ade2-101 met2 lys2-801 his3Δ200 trp1::hisG leu2Δ ura3-52::URA3::TDH3pr-SUS-GFP dfm1Δ::KanMX hrd1Δ::NatR*	This study
SEN 214	Mata *ade2-101::ADE2::URA3::TDH3pr-HMG2-GFP met2 lys2-801 his3Δ200 trp1::hisG leu2Δ ura3-52 dfm1Δ::KanMX*	This study
RYH 10849	Mata *ade2-101::ADE2::URA3::TDH3pr-HMG2-GFP met2 lys2-801 his3Δ200 trp1::hisG leu2Δ ura3-52 dfm1Δ::KanMX CEN::LEU2::prDFM1*	Neal, 2018
SEN 10846	Mata *ade2-101::ADE2::URA3::TDH3pr-HMG2-GFP met2 lys2-801 his3Δ200 trp1::hisG leu2Δ ura3-52 dfm1Δ::KanMX CEN::LEU2::prDER1*	Neal, 2018
RYH 10847	Mata *ADE2::URA3:: TDH3pr-Hmg2-GFP met15Δ0 his3Δ1 leu2Δ0 ura3Δ0 dfm1Δ::KanMX pdr5Δ::NatR CEN::LEU2::pDFM1-5Ashp-3HA*	Neal, 2018
RYH 10848	*Mata ADE2::URA3:: TDH3pr-Hmg2-GFP met15Δ0 his3Δ1 leu2Δ0 ura3Δ0 dfm1Δ::KanMX pdr5Δ::NatR CEN::LEU2::pDER1-SHP-3HA*	Neal, 2018
RYH 10849	Mata *ADE2::URA3:: TDH3pr-Hmg2-GFP met15Δ0 his3Δ1 leu2Δ0 ura3Δ0 dfm1Δ::KanMX pdr5Δ::NatR CEN::LEU2::pDFM1-AA-3HA*	Neal, 2018
RYH 10623	Mata *ADE2::URA3:: TDH3pr-Hmg2-GFP met15Δ0 his3Δ1 leu2Δ0 ura3Δ0 dfm1Δ::KanMX pdr5Δ::NatR CEN::LEU2::pDFM1-A*x_3*A-3HA*	Neal, 2018
RYH 10463	Mata *ade2-101 met2 lys2-801 his3Δ200 trp1::hisG leu2Δ ura3-52 dfm1Δ::NatR CEN::HIS3:PDR5*-HA*	Neal, 2018
SEN198	Mata *ade2-101 met2 lys2-801 his3Δ200 trp1::hisG leu2Δ ura3-52 dfm1Δ::KanMX CEN::URA3::STE6-166-HA-GFP*	This study

SEN 430	Mata *ade2-101 met2 lys2-801 his3Δ200 trp1::hisG leu2Δ ura3-52 dfm1Δ::NatR CEN::URA3::CPY*-GFP CEN::LEU2::DER1-HA*	This study
SEN 410	Mata *ade2-101 met2 lys2-801 his3Δ200 trp1::hisG leu2Δ ura3-52 dfm1Δ::KanMX CEN::URA3::CPY*-GFP CEN::LEU2::DFM1-HA*	This study
SEN 440	Mata *ade2-101 met2 lys2-801 his3Δ200 trp1::hisG leu2Δ ura3-52::URA3::TDH3pr-SUS-GFP dfm1Δ::KanMX CEN::URA3::LEU2::ADH1pr-DERLIN-1-MYC*	This study
SEN 441	Mata *ade2-101 met2 lys2-801 his3Δ200 trp1::hisG leu2Δ ura3-52::URA3::TDH3pr-SUS-GFP dfm1Δ::KanMX CEN::URA3::LEU2::ADH1pr-DERLIN-2-MYC*	This study

Note: Due to the rapid suppression nature of *dfm1Δ* yeast strains (Bhaduri and Neal, 2021), all *dfm1Δ* strains should be transformed with DFM1 mutant constructs (listed in Table S1) before the start of any experiment.
